# Quantum and Topological Dynamics of GKSL Equation in Camel-like Framework

**DOI:** 10.3390/e27101022

**Published:** 2025-09-28

**Authors:** Sergio Manzetti, Andrei Khrennikov

**Affiliations:** Department of Mathematics, Faculty of Technology, Linnaeus University, 351 95 Växjö, Sweden; sergio.manzetti@fjordforsk.no

**Keywords:** open quantum systems, Lindblad equation (GKSL), von Neumann entropy, camel-like entropy behavior, quantum decoherence and stability

## Abstract

We study the dynamics of von Neumann entropy driven by the Gorini–Kossakowski–Sudarshan–Lindblad (GKSL) equation, focusing on its camel-like behavior—a hump-like entropy evolution reflecting the system’s adaptation to its environment. Within this framework, we analyze quantum correlations under decoherence and environmental interaction for three sets of quantum states. Our results show that the sign of the entanglement entropy’s derivative serves as an indicator of the system’s drift toward either classical or quantum information exchange—an insight relevant to quantum error correction and dissipation in quantum thermal machines. We parameterize quantum states using both single-parameter and Bloch-sphere representations, where the angle θ on the Bloch sphere corresponds to the state’s position. On this sphere, we construct gradient and basin maps that partition the dynamics of quantum states into stable and unstable regions under decoherence. Notably, we identify a *Braiding ring* of decoherence-unstable states located at $\theta =\frac{3\pi }{4}$; these states act as attractors under a constructed Lyapunov function, illustrating the topological and dynamical complexity of quantum evolution. Finally, we propose a testable experimental setup based on camel-like entropy and discuss its connection to the theoretical framework of this entropy behavior.

## 1. Introduction

We study the open-system dynamics of the von Neumann entropy driven by the Gorini–Kossakowski–Sudarshan–Lindblad (GKSL) equation, with particular interest in its so-called camel-like behavior—a hump-shaped evolution of entropy that reflects the system’s adaptation to its surrounding environment. This characteristic profile offers insights into how quantum systems process environmental influences through information-theoretic quantities such as entropy.

In order to analyze interactions between two or more elements in a quantum information system, we represent these elements or events as states of information, which we term *infons*. While the term *infon* has appeared in various contexts in the literature [1,2,3], here we adopt it to denote fundamental units of quantum information that describe interactions such as those between two bosons, the exchange of cognitive signals between neurons [2], or quantum tunneling of electron pairs across a potential barrier [3]. The dynamics of these interacting elements can be effectively modeled within the framework of open quantum systems [1]. This formalism provides a fully quantum-mechanical description of a system S as it evolves under the influence of a local environment ϵ [4,5,6]. The environment ϵ accounts for effects such as decoherence and dephasing, which impact the state of the system S.

The system-environment relationship can be represented hierarchically as

(1) S⊂ϵ⊂U,

where U represents the highest containing state (e.g., an isolated entangled system subjected to an electromagnetic field).

The evolution of the system S under the influence of ϵ can be described using the density matrix formalism. Let $\rho_{\mathrm{initial}}$ denote the initial state of S. Under the action of a Hamiltonian $\hat{H}$ (representing a local perturbation within ϵ), the system evolves to a final state $\rho_{\mathrm{final}}$:

(2) $$\rho_{\mathrm{initial}}\overset{\hat{H}}{⟶}\rho_{\mathrm{final}}.$$

The environment ϵ can be an electromagnetic field for particles [7,8,9,10] or even a cognitive environment for neuronal cells, including electric currents, neurotransmitters, and other stimuli [11].

### 1.1. Quantum-like Formalism for the GKSL Equation

The foundational framework of open quantum systems can be extended beyond traditional physical scenarios to model quantum-like processes in biological and cognitive systems. Such models have been increasingly applied to represent quantum phenomena across natural sciences [12].

For instance, neuronal signals (treated as infons) can be described by quantum observables and probabilities, where information processing is governed by specific dynamics between decoherence and non-unitary evolution of quantum states. The transition of a neuronal state under environmental perturbations can be represented as a superposition of wavefunctions [6], where the wavefunction of the neuron exposed to some perturbation undergoes the following transition:

(3) $$\psi_{ss}\overset{\hat{H}}{⟶}C_{0}\psi_{ex}+C_{1}\psi_{qs},$$

where $\psi_{ss}$ denotes the wavefunction at steady state with the environment, $\psi_{ex}$ the excited state, and $\psi_{qs}$ the quiescent state. Here, $C_{0}$ and $C_{1}$ are complex coefficients satisfying the normalization condition $|C_{0}{|}^{2}+{\left|C_{1}\right|}^{2}=1$. $\hat{H}$ is a locally acting perturbation within ϵ, and ϵ represents the totality of electrochemical signals in the environment of the neuron, including electric currents, EMF, and neurotransmitters generated by other neurons [13].

In line with quantum theory, the transition induced by $\hat{H}$ in (3) can be represented within the density matrix formalism:

(4) $$\rho_{\mathrm{initial}}\overset{\hat{H}}{⟶}\rho_{\mathrm{final}},$$

where $\rho_{\mathrm{initial}}$ transitions to $\rho_{\mathrm{final}}$, describing the probabilistic nature of superposition. Now, in order to construct the density matrix from a state vector, we define H as a complex Hilbert space, where a pure quantum state is represented by a unit vector |ψ〉∈H, and its dual vector $\langle \psi |\in H^{*}$ by:

(5) 〈ψ|:|ϕ〉↦〈ψ|ϕ〉∈C.

Then, the final density matrix $\rho_{\mathrm{final}}$ corresponds to the pure state described by the outer product:

(6) $$\rho_{\mathrm{final}}=|\psi_{\mathrm{final}}\rangle \langle \psi_{\mathrm{final}}|,$$

with the pure state $\psi_{\mathrm{final}}$ given by

(7) $$|\psi_{\mathrm{final}}\rangle =C_{0}|0\rangle +C_{1}|1\rangle ,$$

and

(8) $$\langle \psi_{\mathrm{final}}|=C_{0}^{*}\langle 0|+C_{1}^{*}\langle 1|,$$

where $C_{0}$ and $C_{1}$ are complex coefficients, and $C_{0}^{*}$ and $C_{1}^{*}$ are their complex conjugates, respectively.

By examining Equation (3), we can see that $\psi_{ex}$ and $\psi_{qs}$ are represented by $\rho_{final}$ in (6), with arbitrary coefficients in both real and complex form. Thus, we have demonstrated how the biological state transition in (3) can be represented by a superposition of quantum states in an open quantum system via (6). This formulation automatically satisfies the Born rule, which gives the probability of measuring the system:

(9) $$P\left(\alpha \right)=|\langle \alpha |\psi_{\mathrm{final}}\rangle {|}^{2}.$$

The time-dependent interaction of such a measurable open quantum system with a specific environment can be studied using the *Gorini–Kossakowski–Sudarshan–Lindblad (GKSL)* equation [14,15,16]. This equation describes how the system stabilizes to a steady state as time approaches infinity [2]:

(10) $$\lim_{t\to \infty }\hat{\rho }\left(t\right)={\hat{\rho }}_{steady}$$

### 1.2. GKSL Operator Structure and Dynamics

Let us first introduce the space of states, which we devise using the finite-dimensional Hilbert space H endowed with the scalar product 〈·,·〉. Let the space of density operators (which represent the states of the system) be denoted by D(H), and the space of all linear operators be given by L(H). Furthermore, L(H) is a complex Hilbert space with the scalar product $\langle A|B\rangle =\mathrm{Tr}\left(A^{†}B\right)$, with *A* and *B* being operators. Furthermore, we have that the environment (i.e., dephasing/decoherence) is represented by superoperators in L(H) that act on the density matrix ρ. The GKSL equation is the time-dependent master equation for open quantum systems, developed in [15,17] from the Schrödinger equation. It is described by

(11) $$\frac{\partial \rho (t)}{\partial t}=-i\left[H,\rho \left(t\right)\right]+L\left[\rho \left(t\right)\right].$$

Here, ρ(t) is the unknown probability density matrix, which evolves with time and is used to resolve properties of an open quantum system, such as entropy, entanglement, and decoherence [15,17]. The first term on the RHS in (11), the Hamiltonian, is the unitary and deterministic part of the evolution, while the second term on the RHS in (11), the dissipation operator, represents the non-unitary effects on the density matrix that ultimately represent non-deterministic evolution of the system. The Hamiltonian represents the internal dynamics of the system at hand, and are often given by the Pauli matrices; however, in some cases, it can also include components related to the interaction with the environment [18]. The dissipation operator, L, is the GKSL superoperator given by

(12) $$L\left[F\left(\rho \right)\right]=\sum_{k}\left(L_{k}\rho L_{k}^{†}-\frac{1}{2}\left\{L_{k}^{†}L_{k},\rho \right\}\right),$$

where $L^{†}$ is the adjoint of the jump operator *L*, defined by the relation:

(13) $$\langle \psi ,L\phi \rangle =\langle L^{†}\psi ,\phi \rangle .$$

This relation holds for any linear operator *L*, Hermitian or not, and defines $L^{†}$ with respect to the inner product. In general, the jump operators *L* in the Lindblad formalism are non-Hermitian, representing dissipative processes such as decoherence or decay. Hence, we have the GKSL equation given by

(14) $$\frac{d\rho }{dt}=-i\left[H,\rho \right]+\gamma \sum_{k}\left(L_{k}\rho L_{k}^{†}-\frac{1}{2}\left\{L_{k}^{†}L_{k},\rho \right\}\right),$$

where γ is the relaxation factor for decoherence processes to the dissipation operator. Each jump operator represents the special type of interaction of the system S by the density matrix ρ in (10), with the environment ϵ (described by L), and vary according to the decoherence and dissipation resulting by the formalism of *H* and L. This forms the main difference between the original Schrödinger equation and the GKSL equation: the GKSL equation produces stabilization of its solution to the steady-state (10).

### 1.3. Camel-like Framework in Entropy Evolution of Open Quantum Systems

In the study of complex systems, entropy serves as a fundamental measure of disorder and information content. We introduce the concepts of *N*-hump camel-like entropy to describe distinct temporal profiles of entropy evolution, building on previous studies [11,12,13]. A one-hump camel-like entropy is characterized by a single local maximum and minimum, followed by damped oscillations, while a two-hump camel-like entropy exhibits two such maxima and minima before stabilizing. These definitions are inspired by the analogy of a camel’s humps, providing an intuitive visualization of entropy dynamics. In quantum information research, these profiles could prove invaluable for analyzing the behavior of quantum systems undergoing decoherence, entanglement dynamics, or information scrambling. For instance, a two-hump entropy profile might indicate the presence of intermediate metastable states in a quantum process, while a one-hump profile could describe simpler, single-phase transitions. Furthermore, we can also obtain N-humps and obtain Rabi-like oscillations [19] in the entropy evolution, which have similarities to the study of spin-state fractions in a single-atom system [20]. By categorizing entropy evolution in this way, we may gain a deeper understanding of the underlying mechanisms governing quantum systems, paving the way for improved control and optimization in quantum computing and communication protocols using the named framework.

**Definition** **1** (Camel-like Behavior of Entropy)**.**

***One-Hump Camel-Like Behavior of Entropy***

*Consider the following time intervals:*

$$I_{1}=\left[0,t_{\max}\right),\;I_{2}=\left(t_{\max},t_{\min}\right),\;I_{3}=\left(t_{\min},+\infty \right)$$

*We characterize these intervals using the derivative of entropy $E^{\prime }\left(t\right)$ as follows:*

*1.*  *$E^{\prime }\left(t\right)>0$ on $I_{1}$, and $E^{\prime }\left(t_{\max}\right)=0$.*
*2.*  *$E^{\prime }\left(t\right)<0$ on $I_{2}$, and $E^{\prime }\left(t_{\min}\right)=0$.*
*3.*  *On $I_{3}$, E(t) exhibits damped oscillations in amplitude.*

*This behavior corresponds to a one-hump camel analogy, where the entropy profile has a single local maximum at $t_{\max}$ and a single local minimum at $t_{\min}$.*

***Two-Hump Camel-Like Behavior of Entropy***

*Consider the following time intervals:*

$$I_{1}=\left[0,t_{\max_{1}}\right),\;I_{2}=\left(t_{\max_{1}},t_{\min_{1}}\right),\;I_{3}=\left(t_{\min_{1}},t_{\max_{2}}\right),\;I_{4}=\left(t_{\max_{2}},t_{\min_{2}}\right),\;I_{5}=\left(t_{\min_{2}},+\infty \right)$$

*We characterize these intervals using the derivative of entropy $E^{\prime }\left(t\right)$ as follows:*

*1.*  *$E^{\prime }\left(t\right)>0$ on $I_{1}$, and $E^{\prime }\left(t_{\max_{1}}\right)=0$.*
*2.*  *$E^{\prime }\left(t\right)<0$ on $I_{2}$, and $E^{\prime }\left(t_{\min_{1}}\right)=0$.*
*3.*  *$E^{\prime }\left(t\right)>0$ on $I_{3}$, and $E^{\prime }\left(t_{\max_{2}}\right)=0$.*
*4.*  *$E^{\prime }\left(t\right)<0$ on $I_{4}$, and $E^{\prime }\left(t_{\min_{2}}\right)=0$.*
*5.*  *On $I_{5}$, E(t) exhibits damped oscillations in amplitude.*

*This behavior corresponds to a two-hump camel analogy, where the entropy profile has two local maxima at $t_{\max_{1}}$ and $t_{\max_{2}}$, and two local minima at $t_{\min_{1}}$ and $t_{\min_{2}}$.*

**N-hump Camel-like Behavior of Entropy**

Let E(t) be the entropy of a quantum system evolving under the GKSL equation. We define E(t) as having *N humps* (local maxima) if there exists a sequence of critical points $\left\{t_{\max_{1}},t_{\min_{1}},\ldots ,t_{\max_{N}},t_{\min_{N}}\right\}$ such that the time domain $R^{+}$ is partitioned into 2N+1 intervals:

$$\begin{array}{cc} I_{0} & =[0,t_{\max_{1}}),\\ I_{2k-1} & =(t_{\min_{k-1}},t_{\max_{k}})\;\mathrm{for}\;k=1,\ldots ,N,\\ I_{2k} & =(t_{\max_{k}},t_{\min_{k}})\;\mathrm{for}\;k=1,\ldots ,N,\\ I_{2N+1} & =(t_{\min_{N}},+\infty ), \end{array}$$

where $t_{\min_{0}}\equiv 0$, and the entropy derivative $E^{\prime }\left(t\right)$ satisfies the following:

1. **Growth Phases**: $E^{\prime }\left(t\right)>0$ on $I_{2k-1}$ for all *k*, with $E^{\prime }\left(t_{\max_{k}}\right)=0$ (local maxima).
2. **Decay Phases**: $E^{\prime }\left(t\right)<0$ on $I_{2k}$ for all *k*, with $E^{\prime }\left(t_{\min_{k}}\right)=0$ (local minima).
3. **Asymptotic Damping**: On $I_{2N+1}$, E(t) exhibits underdamped oscillations converging to a steady-state value $E_{\mathrm{ss}}$.

From this definition, we see that the defined camel-like entropy profiles, characterized by transient maxima and minima in disorder, evoke parallels to barrier-crossing phenomena across diverse systems, based on their number of humps. For instance, in enzyme catalysis [21], the one-hump entropy profile mirrors the transient disordered transition state (hump) that precedes product stabilization—akin to overcoming a single entropic barrier during catalysis. In contrast, two-hump entropy aligns with multi-phase processes like protein folding [22], where disordered intermediates and misfolded states generate sequential entropy peaks, or quantum tunneling [23] with successive decoherence events. Moreover, spin-fraction measurement for single-atom systems [20] shows Rabi oscillations, which are in a damped form here described as N-hump camel-like behavior.

### 1.4. Operators

Now that we have formalized the definition of the camel-like entropy, we can define the Hamiltonian *H* and the dissipation operator L that governs the dynamics of the system following the camel-like formalism [2,11,13]. By the GKSL equation:

(15) $$\rho^{\prime }\left(t\right)=-i\left[H,\rho \left(t\right)\right]+\gamma \left(C\rho \left(t\right)C^{†}-\frac{1}{2}\left\{C^{†}C,\rho \left(t\right)\right\}\right),$$

we have, γ which is the relaxation rate representing the strength of interaction between a quantum system and its environment. We present the model example of the order-stable dynamics that matches the biological order-preserving behavior and select the Hamiltonian *H* and the interaction operator *C* as follows [2]:

(16) $$H=\sigma_{x}=\left(\begin{array}{cc} 0 & 1\\ 1 & 0 \end{array}\right),\;C=\left(\begin{array}{cc} 0 & 1\\ 0 & 0 \end{array}\right).$$

We calculate these operators into their four-dimensional form:

(17) $$H=\sigma_{x}⊗\sigma_{x}=\left(\begin{array}{cccc} 0 & 0 & 0 & 1\\ 0 & 0 & 1 & 0\\ 0 & 1 & 0 & 0\\ 1 & 0 & 0 & 0 \end{array}\right)$$

The dissipation operator redimensioned to N=4 gives the collapse operator *C*, which we extend to act on the two-qubit system. By choosing an operator that acts locally on one qubit, specifically $C=\sigma_{+}⊗I$, where $\sigma_{+}$ is the *raising* operator for the first qubit and *I* is the identity matrix for the second qubit, we obtain

(18) $$C=\sigma_{+}⊗I=\left(\begin{array}{cccc} 0 & 1 & 0 & 0\\ 0 & 0 & 0 & 0\\ 0 & 0 & 0 & 1\\ 0 & 0 & 0 & 0 \end{array}\right).$$

Based on the analysis conducted in [24], we set the relaxation factor $\gamma =\frac{1}{2}$.

Having set the premises for the study of the camel-like framework of the GKSL equation applied to a set of quantum states, we now define our aims. The first aim is to study the evolution of the von Neumann entropy for the set of selected quantum states, and the entanglement entropy and its rate of change, so that we show the performance of the camel-like framework on pure states, product states, and mixed states. Second, we wish to analyze the properties of the camel-like framework by using a gradient field and topological basin analysis, so that we can study the evolution of solutions on the Bloch sphere and their stability. Using this combination of analytical tools, we thus aim to present how the camel-like framework works and to which physical experiment it has strong similarities to.

## 2. Methods of Analysis

### 2.1. State Representation

We can form the given (normalized) states for the three classes of quantum states, pure states, product states, and mixed states: The states of interest are represented thus by the three categories:

**Bell states.**

We include two Bell states in our calculations, namely $\Phi^{+}$ and $\Phi^{-}$.

**Product states.**

Consider the two-qubit state

(19) $$|\psi_{\mathrm{prod}}\rangle =\sqrt{\frac{3}{5}}|00\rangle +\sqrt{\frac{3}{20}}|01\rangle +\sqrt{\frac{1}{5}}|10\rangle +\sqrt{\frac{1}{20}}|11\rangle .$$

This state can be expressed explicitly as a tensor product of two single-qubit states:

(20) $$|\psi_{\mathrm{prod}}\rangle =\left(\sqrt{\frac{3}{5}}|0\rangle +\sqrt{\frac{1}{5}}|1\rangle \right)⊗\left(|0\rangle +\frac{1}{2}|1\rangle \right).$$

Similarly, for the second state

(21) $$|\psi_{\mathrm{prod}}^{\prime }\rangle =\frac{\sqrt{6}}{6}|00\rangle +\frac{\sqrt{6}}{6}|01\rangle +\frac{\sqrt{3}}{3}|10\rangle +\frac{\sqrt{3}}{3}|11\rangle ,$$

we write it explicitly as a tensor product:

(22) $$|\psi_{\mathrm{prod}}^{\prime }\rangle =\left(\frac{1}{\sqrt{6}}|0\rangle +\frac{1}{\sqrt{3}}|1\rangle \right)⊗\left(|0\rangle +|1\rangle \right).$$

**Mixed states.**

The mixed states are defined with probabilities p=q=0.5 using the pure states:

(23) $$\begin{array}{cc} \rho_{1} & =p\rho_{\psi_{3}}+\left(1-p\right)\rho_{\psi_{4}}\\ \; & =0.5|\psi_{3}\rangle \langle \psi_{3}|+0.5|\psi_{4}\rangle \langle \psi_{4}| \end{array}$$

(24) $$\begin{array}{cc} \rho_{2} & =q\rho_{\psi_{5}}+\left(1-q\right)\rho_{\psi_{6}}\\ \; & =0.5|\psi_{5}\rangle \langle \psi_{5}|+0.5|\psi_{6}\rangle \langle \psi_{6}| \end{array}$$

where

(25) $$\begin{array}{cc} |\psi_{3}\rangle & =\frac{1}{\sqrt{2}}|00\rangle +\frac{1}{\sqrt{2}}|01\rangle ,\\ \rho_{\psi_{3}} & =|\psi_{3}\rangle \langle \psi_{3}| \end{array}$$

(26) $$\begin{array}{cc} |\psi_{4}\rangle & =|10\rangle ,\\ \rho_{\psi_{4}} & =|\psi_{4}\rangle \langle \psi_{4}| \end{array}$$

(27) $$\begin{array}{cc} |\psi_{5}\rangle & =|11\rangle ,\\ \rho_{\psi_{5}} & =|\psi_{5}\rangle \langle \psi_{5}| \end{array}$$

(28) $$\begin{array}{cc} |\psi_{6}\rangle & =\frac{\sqrt{3}}{2}|00\rangle +\frac{1}{2}|10\rangle ,\\ \rho_{\psi_{6}} & =|\psi_{6}\rangle \langle \psi_{6}| \end{array}$$

### 2.2. Numerical Calculations

We employ numerical methods to solve the Lindblad equation, specifically utilizing matrix exponential techniques, where we use the forward-Euler method [25] for solving the Lindblad equation to calculate entropy. The calculation of von Neumann entropy uses logarithm base 2 to align with information theory, where entropy measures uncertainty in bits. When we calculate the entanglement entropy, we use the mesolve method as part of the qutip package [26]. Finally, for the calculations of the CHSH parameter, we use the Runge–Kutta method of fourth order. These approaches allow us to study the evolution of the density matrix through the matrix exponential. We analyze the numerical solutions for the density matrices over time to observe the evolution of the mentioned quantum relations as a result of the Lindblad dynamics. All simulations are carried out with Python v3.

### 2.3. Quantum Correlations Computational Code

We form Python codes for the study of the time evolution of the von Neumann entropy for the considered pair of states (Bell states, product states, and mixed states). All states are represented as density matrices, computed as the outer products of the states with their conjugates. The Hamiltonian governing the system is defined as $H=\sigma_{x}⊗\sigma_{x}$, where $\sigma_{x}$ is the Pauli-X matrix. The collapse operator is defined as $C=\sigma_{+}⊗I$, with $\sigma_{+}$ being the raising operator for a single qubit and *I* the identity matrix. The evolution of the system is modeled using the Lindblad equation, which accounts for both the Hamiltonian and dissipative processes. The Lindblad evolution step is implemented by iterating over small time steps Δt, updating the density matrix at each step, and normalizing the result to ensure it remains a valid density matrix. The von Neumann entropy is computed at each time step using the eigenvalues of the density matrix. To avoid numerical issues, eigenvalues are clipped to a small value before taking their logarithm. The time evolution is simulated for 1000 steps, with a time step of Δt=0.01.

#### 2.3.1. Von Neumann and Entanglement Entropy

The entanglement entropy is computed using the von Neumann entropy, which quantifies the amount of quantum entanglement in a system. In the code, we compute this as

$$S\left(\rho \right)=-\mathrm{Tr}\left(\rho \log_{2}\rho \right)$$

where the logarithm is taken in base 2, and the trace operation sums over the eigenvalues of the matrix. To calculate the entanglement entropy for the two-qubit systems, the following procedure is applied in the Python code:

1. Partial Trace: The first step is to compute the partial trace over one of the qubits (in this case, the second qubit) to obtain the reduced density matrix for the first qubit. The partial trace operation effectively “traces out” the second qubit, leaving a reduced matrix that describes the subsystem of interest. For a two-qubit system described by the density matrix ρ, the partial trace over the second qubit is computed as:

$$\rho_{\mathrm{red}}={\mathrm{Tr}}_{2}\left(\rho \right)$$

For the two-qubit Bell states, this results in a 2 × 2 matrix representing the reduced density matrix of the first qubit.

2. Eigenvalue Calculation: After obtaining the reduced density matrix, its eigenvalues are computed. These eigenvalues represent the probabilities associated with the possible states of the reduced system.

3. Von Neumann Entropy: Once the eigenvalues of the reduced density matrix are obtained, the von Neumann entropy is computed by applying the following formula:

$$S\left(\rho_{\mathrm{red}}\right)=-\sum_{i}\lambda_{i}\log_{2}\left(\lambda_{i}\right)$$

where $\lambda_{i}$ are the eigenvalues of the reduced density matrix $\rho_{\mathrm{red}}$. To prevent numerical issues, the eigenvalues are clipped to a small value before taking the logarithm (e.g., $\lambda_{i}\geq 10^{-14}$) to avoid computing $\log_{2}\left(0\right)$. This process is repeated for each time step during the evolution, allowing us to track how the entanglement entropy changes over time due to the effects of the dissipative dynamics introduced by the Lindblad evolution.

#### 2.3.2. Derivative of the Entanglement Entropy

The first derivative of the entanglement entropy with respect to time $\frac{dS_{A}}{dt}$ is computed numerically using the ‘np.gradient’ function on the entanglement entropy values, which are computed as in the von Neumann entropy code.

### 2.4. Gradient Map Analysis of the $S^{2}$ Manifold Represented by the Bloch Sphere

The gradient flow analysis was implemented in MATLAB 2025a through a custom script that visualizes critical dynamics on the Bloch sphere representation of quantum states. The script defines the mathematical components $g_{1}\left(\theta \right)=\left(\cos\theta +\sqrt{2}/2\right)\left(\cos\theta +1\right)$ and $g_{2}\left(\theta ,\phi \right)=\sin^{2}\phi \sin^{2}\left(\phi -\pi /2\right)\left(\cos\theta +1\right)$ from (53), along with the squared magnitude function $h\left(\theta ,\phi \right)=g_{1}^{2}+g_{2}^{2}$ whose gradient drives the flow analysis. Using numerical differentiation with a delta of $10^{-6}$, the gradient is computed and incorporated into a differential equation system modeling the negative gradient flow, solved via MATLAB’s ode45 Runge–Kutta solver with high precision tolerances ($10^{-6}$). The Bloch sphere is rendered as a surface mesh colored by the *z*-coordinate, with the southern quarter-circle at θ=3π/4 explicitly highlighted in red as the critical manifold. Six unstable initial states are marked with distinct colors and enlarged markers, representing the singularities in (48) where the map $f:S^{2}\to R^{2}$ is undefined (two of the six states overlap on the South Pole—hence yielding five visible states as points on the Bloch sphere). White gradient flow vectors are superimposed on the sphere using normalized arrows, and cyan trajectories from the unstable points visualize convergence behavior toward stable attractors. The analysis includes seven viewing angles, including a dedicated South Pole view, along with temporal evolution plots of the *h*-function to demonstrate the system’s dynamical stability and convergence properties around the critical southern ring, where states share a common instability towards decoherence.

### 2.5. Topological Basin Analysis on the $S^{2}$ Manifold Represented by the Bloch Sphere

The MATLAB script for analyzing the topological dynamics of the GKSL equation simulates gradient flows on a spherical coordinate grid (θ,φ) across the Bloch sphere, with the singularities in the set (48) corresponding to the zeros of the functions $g_{1}$ and $g_{2}$. The system evolves according to the negative gradient of the scalar function $h\left(\theta ,\varphi \right)=g_{1}{\left(\theta \right)}^{2}+g_{2}{\left(\theta ,\varphi \right)}^{2}$, where $g_{1}\left(\theta \right)=\left(\cos\theta +\sqrt{2}/2\right)\left(\cos\theta +1\right)$ and $g_{2}\left(\theta ,\varphi \right)=\sin^{2}\varphi \sin^{2}\left(\varphi -\pi /2\right)\left(\cos\theta +1\right)$. The singularities occur where both $g_{1}=0$ and $g_{2}=0$: at the North Pole (θ=0), the South Pole (θ=π), and along the critical latitude θ=3π/4 (where $\cos\theta =-\sqrt{2}/2$). The gradient flow equations incorporate regularization to handle the coordinate singularity at the poles. Trajectories are integrated via MATLAB’s ode45 solver from initial conditions sampled on a 30×60 grid, and classified into three basins: (1) the northern hemisphere (θ<3π/4), (2) the South Pole region (3π/4≤θ≤π), and (0) elsewhere. Visualizations in both 2D spherical coordinates and 3D Cartesian projections explicitly mark these singularities and basin boundaries, with multiple viewing angles emphasizing the southern hemisphere structure to reveal the system’s global topological landscape.

## 3. Results and Discussion

### 3.1. Entropy for Bell States, Product States, and Mixed States

The numerical calculations allow us to calculate the most fundamental property of the system, defined by the von Neumann entropy S(ρ). The von Neumann entropy (also defined as quantum entropy) quantifies the uncertainty of a quantum state represented by the density operator ρ, defined as:

(29) S(ρ)=−Tr(ρlogρ).

**Figure 1:**
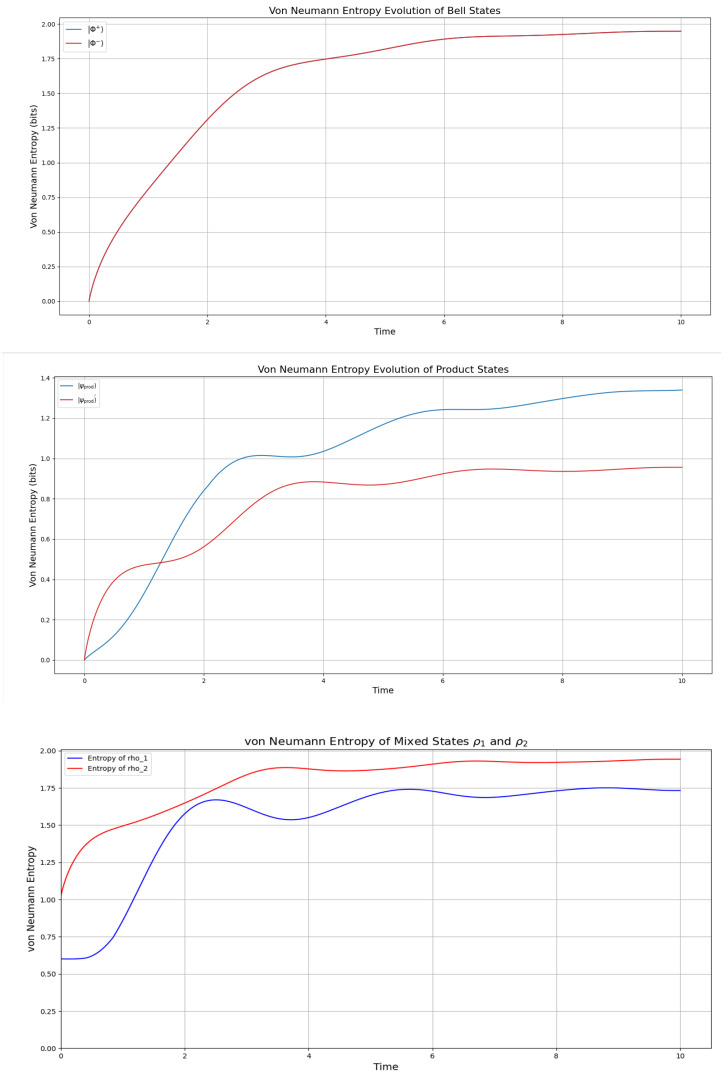
Entropy of the Bell states $\Phi^{+},\Phi^{-}$ (top—overlapping to one plot), the product states in (19) and (21) (middle), and the mixed states in (23) (with p=0.5 and q=0.5), calculated by the Lindbladian evolution in the GKSL framework given in [2] with $\gamma =\frac{1}{2}$. The simulation was initiated with all pairs of states entangled (not separated) at t=0.

Following Figure 1, both Bell states $|\phi^{+}\rangle$ and $|\phi^{-}\rangle$ initially display an entropy of approximately zero, indicating that the system starts in a pure state. Over time, the entropy increases to about 1.85 bits, suggesting a process of mixing, likely due to decoherence. Notably, the identical evolution of these states implies that the dynamics are phase-insensitive, meaning that the system’s evolution does not depend on the specific phase relationship between the states. For the product states $|\psi_{prod}\rangle$ and $|\psi_{prod}^{\prime }\rangle$, the initial entropy of the full system is also zero, but the reduced entropies are approximately 0.72 bits and 0.97 bits, respectively. As the system evolves, the entropy increases, reaching values of 1.3 bits and 0.8 bits. This growth in entropy suggests that entanglement is generated over time, transforming the initially separable states into partially entangled states. The differences in the entropy values indicate that the two product states undergo different degrees of entanglement formation, possibly due to variations in their initial superposition coefficients.

For the mixed states $\rho_{1}$ and $\rho_{2}$, the initial reduced entropies are 1 bit and approximately 0.65 bits. The entropy of $\rho_{1}$ fluctuates between 0.75 and 1.75 bits before stabilizing at 1.6 bits, while $\rho_{2}$ reaches a peak of 1.9 bits and settles there. This suggests more complex dynamical behavior compared with the Bell and product states, possibly due to the probabilistic mixture of pure states in their initial configurations. The long-term stabilization of entropy values indicates that the system reaches a steady state, reflecting a balance between entanglement generation and dissipation effects. Overall, all simulations generate steady states with a maximal value of S≈1.85, it is, however, noteworthy that the interval t∈[0,2] represents a transition phase with decoherence and coherence, which generate one-hump camel-like entropy behavior, particularly of the product and mixed states. The Bell states display a lesser undulation in this interval, which, however, is more pronounced in the entanglement entropy seen in the next section.

Regarding the entropy plots (see Figure 1), we observe a striking similarity to the spin-up electron fraction measured for a single phosphorus donor atom in silicon subjected to microwave radiation, as reported by Pla et al. [20], however, with a considerable damping effect. Specifically, their results show Rabi oscillations of the spin-up fraction, which we find mirrored in the non-monotonic time evolution of the von Neumann entropy in our system for both product states (ψ, $\psi^{\prime }$) and mixed states ($\rho_{1}$, $\rho_{2}$). For the product states in Figure 1, particularly $\psi^{\prime }$ and $\rho_{1}$, we observe the entropy behavior as “camel-like” due to the characteristic rise, dip, and subsequent increase, resembling a double-humped structure in the entropy curves. Indeed, for the mixed states in Figure 1, both $\rho_{1}$ and $\rho_{2}$ exhibit a rise-dip pattern, with $\rho_{1}$ (blue curve) showing a more pronounced “camel-like” shape, 1.75 bits, while $\rho_{2}$ (red curve) reaches a higher entropy of 1.85 (as the Bell states) bits with a subtler dip. This non-monotonic pattern in both cases can be attributed to coherent dynamics driven by noncommuting terms in the Hamiltonian, which induce damped Rabi oscillations [9,19]. These oscillations cause the qubit’s state to evolve coherently, leading to a transient oscillatory profile in the entropy when traced over a subsystem; however, weak dissipation, as introduced by the dissipation term γL(ρ), damps these oscillations after a single cycle, resulting in the observed “camel-like” shape before the entropy stabilizes at a steady value.

**Figure 2:**
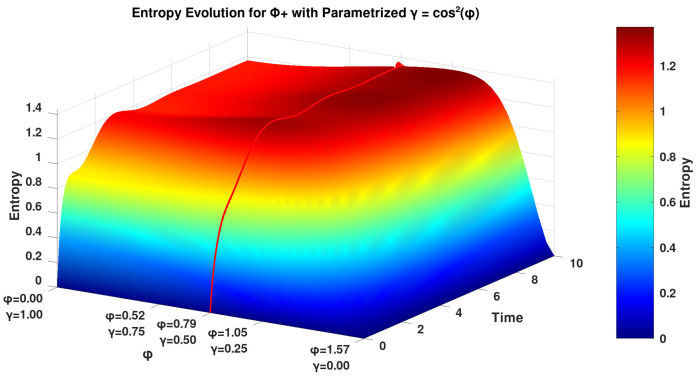
Entropy evolution of the Bell state $\Phi^{-}$ calculated by the GKSL equation under the framework given in [2] with $\gamma =\cos^{2}\phi$. *x*-axis: ϕ, *y*-axis: Time, *z*-axis: Entropy plot in red: Entropy of the system by the inflection point value of γ=0.5 derived from the analysis by [24], corresponding to the equivalent plot in Figure 1 (generated in Octave/Python) for optimal graphics. We only plot $\Phi^{-}$ since $\Phi^{+}$ is equal.

We add here an additional analysis of the evolution of the entropy for the Bell states using a parameterized coefficient of γ. We parameterize $\gamma =\cos^{2}\phi$ on the domain ϕ∈[0,π/2]. By this, we obtain a map of the effect of the relaxation factor γ on the unit interval, providing a smooth variation for the entropy evolution analysis, which is shown in Figure 2.

We find an interesting result in the plot of the parameterized under the framework given in [2] with relaxation factor γ (Figure 2), where the maximal number of undulations in the entropy (between 1 and 2 bits) occurs for ϕ values approximately between $\phi \approx 1.0\approx \frac{1}{3}\pi$ radians and $\phi \approx 1.2\approx \frac{2}{5}\pi$ radians. The special behavior of the entropy observed in Figure 2 is aligned with the damped Rabi oscillations detected for spin-fractions of single silicon atoms studied by Pla et al. [20]. In this study, several different spin oscillations were observed after various input powers were applied to a single silicon atom device. The plots of these different spin oscillations result as highly similar plots to the various plots represented in Figure 2, indicating that the Khrennikov setup of the GKSL equation may be suitable for the calculation of properties of such spin-fractions from single-atom systems and entropy properties therein.

The Rabi oscillations we observe in Figure 2 signify intermittent entanglement degradation and revival, arising from the competition between the Hamiltonian’s coherent dynamics and the dissipative action of the $\sigma_{+}⊗I$ operator. Furthemore, the undulations in the entropy evolution for $|\Phi^{-}\rangle$ under $\gamma =\cos^{2}\phi$ are signatures of non-Markovian dynamics [27], violating the Markovian monotonicity condition $\partial_{t}S\left(t\right)\geq 0$ where these oscillations lead to an increasing number of metastable entanglement revival via Liouvillian exceptional points [28]. These points are degeneracies in quantum open systems (and classical systems as well) and have significant relevance to physics and optics [28]. Such exceptional points define an entropy evolution where two or more eigenvalues coalesce [29] and thus revoke that Φ-type Bell states could be optimally protected against external influence (i.e., decoherence channels) when γ = 0.1–0.3, making them promising for quantum devices to reduce noise and error.

### 3.2. Entanglement Entropy

**Figure 3:**
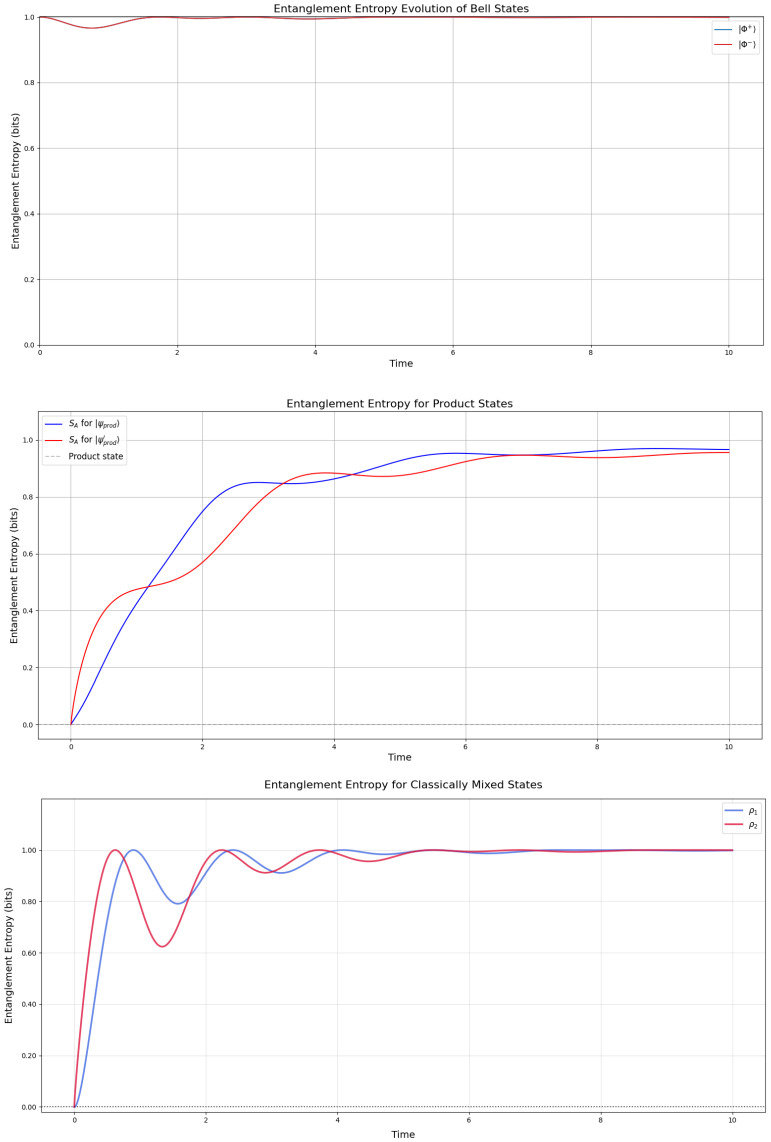
Entanglement entropy of the Bell states (which overlap to one plot) $\Phi^{+},\Phi^{-}$ (top), the product states in (19) (middle), and the mixed states in (23) (with p=0.5 and q=0.5) (bottom), calculated by the Lindbladian evolution in the GKSL framework given in [2] with $\gamma =\frac{1}{2}$. The plots of the Bell states overlap, while for the other states, they show each individual reduced density matrix entropy under the GKSL evolution. The simulation was initiated with all pairs of states entangled (not separated) at t=0.

The entanglement entropy is defined by the von Neumann entropy of the reduced density matrix and is employed by being computed for one of the states, working as a key metric for understanding the coherence and mixedness of quantum states over time. Following the top plot in Figure 3, we focus on the Bell states $|\Phi^{+}\rangle =\frac{1}{\sqrt{2}}(|00\rangle +\left|11\rangle \right)$ and $|\Phi^{-}\rangle =\frac{1}{\sqrt{2}}(|00\rangle -\left|11\rangle \right)$, both initially maximally entangled with S(A)=1 (due to normalization, scaling to a maximum value of 1 for clarity). As time progresses, the entropy (represented by a single red curve, as both states evolve identically) dips slightly around t=1, reflecting the dissipative influence of the raising operator, which nudges the system toward the ground state |00〉. However, the entropy stabilizes near 1, significantly above zero, indicating robust residual entanglement. This persistence suggests that the Hamiltonian *H*, by facilitating transitions between |00〉 and |11〉, effectively counteracts the disentangling effects of dissipation, preserving quantum correlations in the long-time limit.

For the product states $|\psi_{prod}\rangle$ and $|\psi_{prod}^{\prime }\rangle$, the entanglement entropy dynamics reveal three distinct phases. Initially, both states show zero entanglement entropy (as expected for separable states), followed by a sharp increase to approximately 0.8–0.9 bits, indicating significant entanglement generation under the system’s dynamics. The subsequent plateau around 1 bits demonstrates stable entanglement preservation, with the states remaining highly (though not maximally) entangled. The close similarity between both curves suggests the dynamics affect different product states in qualitatively similar ways, while the slight difference in their steady-state values may reflect varying degrees of correlation in their initial configurations. The mixed states $\rho_{1}$ and $\rho_{2}$ (Figure 3) exhibit more complex behavior, following a damped Rabi oscillation mode, beginning with zero entanglement despite their mixed nature, which highlights their preparation using mixtures of product states. Both states rapidly develop entanglement, with both reaching maximal entanglement (1 bit) and also dipping like the Bell states in the interval I=[1,2]. This non-monotonic evolution suggests competing dynamics with initial entanglement generation through unitary evolution followed by partial disentanglement, due to decoherence or specific Hamiltonian terms. We pay particular attention to the interval I=[0,2], which aligns with the definition of one-hump in Definition 1, and study it further by the rate of entanglement entropy.

#### Relevance with Established Literature

Our findings can be placed in the broader context of quantum many-body out-of-equilibrium physics, where entanglement entropy is routinely employed as a diagnostic of dynamical phases and information spreading. In particular, global and local quenches in one-dimensional lattice and conformal field theory models produce an initially linear growth of entanglement followed by saturation in finite systems [30,31]. The non-monotonic (“camel-like”) profiles and revivals we observe under GKSL dynamics have analogues in finite spin chains and quenched CFTs, where coherent quasiparticle propagation and finite-size reflections generate oscillations of the entropy [32]. Furthermore, the entropy oscillations and transient entanglement revivals reported here are consistent with signatures of non-Markovian dynamics and information backflow identified in open-system studies [27,33]. Taken together, these observations indicate that the camel-like GKSL framework offers a complementary perspective to the large body of work on quenched lattice/QFT models: it bridges master-equation approaches to entropy dynamics with the many-body literature on quenches, revivals, and memory effects, and thus may help to translate phenomena observed in closed interacting systems into experimentally relevant, dissipative settings.

### 3.3. Maximal Rate of Entanglement Entropy as a Marker of Information Exchange Transitions

Extended results on the evolution of mutual information (see Appendix A) for these quantum systems show that the Lindblad framework described in Section 1.3 generally drives systems toward classical-like information exchange. Nevertheless, in all quantum correlations we report here, we identify a key transition state for $|\psi_{prod}\rangle$, $|\psi_{prod}^{\prime }\rangle$, $\rho_{1}$, and $\rho_{2}$, which corresponds to an interval where the rate of change in entanglement entropy is maximized. This rate is given by

(30) $$\frac{dS_{A}}{dt}=-\mathrm{Tr}\left(\frac{d\rho_{A}}{dt}\left(\log\rho_{A}+1\right)\right),$$

where *A* represents the reduced state of $|\psi_{prod}\rangle$, $|\psi_{prod}^{\prime }\rangle$, $\rho_{1}$, or $\rho_{2}$. The maximal rate of information exchange is thus defined as

(31) $$\mathrm{maximal}\;\mathrm{rate}\;\mathrm{of}\;\mathrm{information}\;\mathrm{exchange}=\max_{\lambda }\left(\frac{dS_{A}}{dt}\right).$$

**Figure 4:**
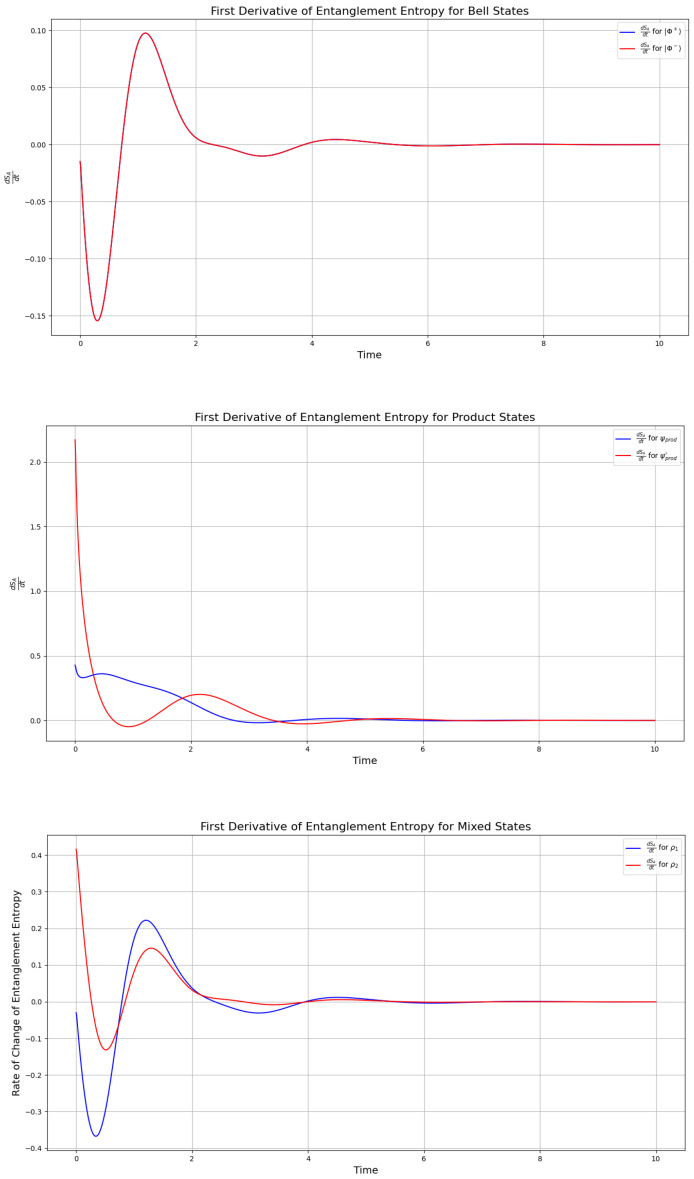
Rate of information exchange for the Bell states (top—overlapping to one plot) $\Phi^{+},\Phi^{-}$ by the first derivative of the entanglement entropy, the product states in (19) (middle), and the mixed states (bottom) in (23) (with p=0.5 and q=0.5), calculated by the first derivative of the entanglement entropy, under the Lindbladian evolution with the camel-like behavior given in [2], with $\gamma =\frac{1}{2}$. The simulation was initiated with all pairs of states entangled (not separated) at t=0.

This period of maximal rate corresponds to the largest increase in entanglement entropy, contrasting with the overall decrease driven by dissipation. The plot of $\frac{dS_{A}}{dt}$ is shown in Figure 4 and highlights this transition. Notably, as we report in an extended version of this study in [13], the mutual information also peaks around t∈[1,2], which is a period contained in the one-hump interval defined in Definition 1, and defined in Definition 1. Thus, we observe that the transition state ($f(x_{b})$) from Definition 1 corresponds to the maximum of the first derivative of the entanglement entropy for all states. This is particularly evident in the rate of change in the information exchange for the Bell states (Figure 4), which exhibit only a minor “hump” in the entanglement entropy within the time interval t∈[1,2] (see Figure 3). This feature is reflected in Figure 4 as an immediate maximum followed by a minimum in the rate of change in the entanglement entropy.

The maximum in the derivative highlights the period t∈[1,2] as the interval of the most rapid rate of information exchange across all simulated systems. This suggests that the maximum rate of change in the entanglement entropy serves as a key indicator for identifying whether the information exchange is transitioning toward classical or quantum-like behavior: if it is classical, the rate of change decreases, while a quantum-like exchange is characterized by a maximum in this rate. This behavior is further supported by the mutual information, which decreases more slowly for the Bell states during this period (see calculation of the mutual information in the Appendix A). Thus, calculating the rate of change in entanglement entropy provides a critical method for predicting whether a system is evolving from classical to quantum information exchange, or vice versa.

Notably, quantum correlations alone often fail to determine the direction of this transition; instead, they primarily indicate whether the system currently exhibits classical or quantum-like behavior. In contrast, when the entanglement entropy can be resolved, its rate of change robustly predicts the system’s evolution toward either regime. In Figure 4, we observe that the transition state $f(x_{b})$ from Definition 1 corresponds to the maximum of the first derivative of the entanglement entropy for all states. This is especially evident in the Bell states, which display a minor “hump” in the entanglement entropy within t∈[1,2] (see Figure 3). Correspondingly, Figure 4 shows an immediate maximum followed by a minimum in the rate of change. These findings are consistent with recent studies [34] demonstrating that quantum-to-classical transitions arise from the dynamical properties of systems in Hilbert space, rather than from operator non-commutativity, as reflected in our calculations.

### 3.4. Quantum Discord

**Figure 5:**
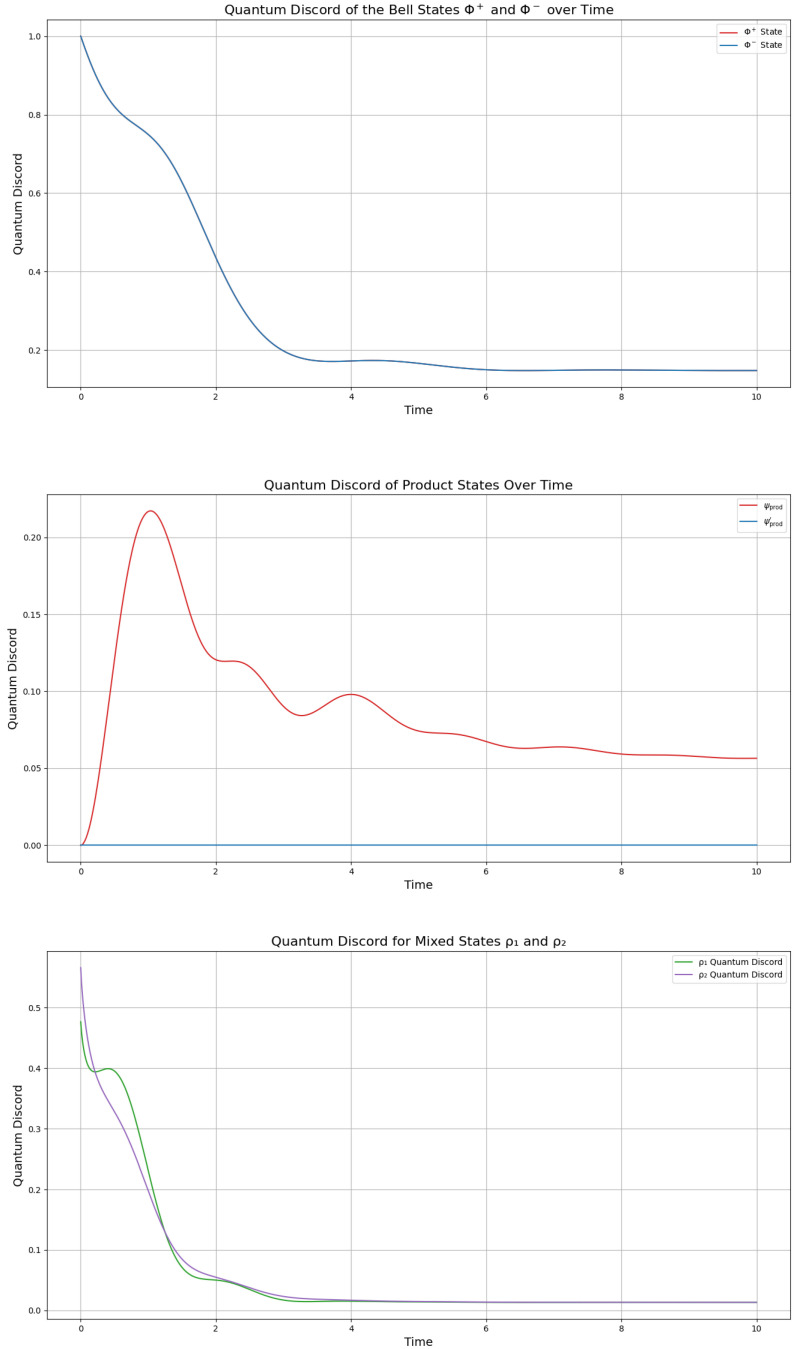
Quantum discord of the states modeled by the Lindbladian evolution in the KB-picture [2], for the Bell states (top—overlapping to one plot), the product states in (19) (middle), and the mixed states in (23) (bottom) with p=0.5 and q=0.5 assigned as probabilities, with $\gamma =\frac{1}{2}$. The simulation was initiated with all pairs of states entangled (not separated) at t=0.

Quantum discord gives a measure of the level of “quantumness” of a system, hence the degree to which the behavior of the qubits is classical or quantistic. This is important to be determined, since the relationship between “quantumness” and quantum relations is not always as expected. For instance, a system can behave classically, but still be entangled, which indicates that quantum discord is an important property of systems and can give better classifications of their behavior in an open quantum system simulation. We calculate and plot the quantum discord of our model states, the Bell states, the product states in (19), (21), and the mixed states in (23), (24) by the Khrennikov-picture, using the Hamiltonians and dissipation operators given by (16) (Figure 5). In this figure, we see that we obtain a stabilization of the quantum discord of the Bell states to a stable steady state after experiencing a small “hump” at the beginning of the simulation. The fact that the Bell states start from quantum discord of *D* = 1 is correct as quantum discord measures non-classical correlations in mixed states, which Bell states initially are not. Clearly, the Bell states lose coherence and their correlation turns into a classical correlation, to a higher extent, by attaining a steady state with a discord of *D* = 0.1. It is noteworthy that the decrease in discord is showing a “hump” during the transition state period of t∈[1,2], as well as for the previous correlations and as defined by Definition 1.

The quantum discord evolution in Figure 5 reveals distinct dynamics for the entangled ($|\psi_{\mathrm{prod}}\rangle$) and separable ($|\psi_{\mathrm{prod}}^{\prime }\rangle$) states. The entangled state displays initial discord D=0 bits, confirming to non-classical correlations, which suddenly increase to D≈0.2 bits in the interval t∈[1,2], and by t=10, it stabilizes to 0.05 bits. In contrast, the separable state maintains D=0 throughout, as expected for a classically correlated system. Notably, the non-zero steady-state discord for $|\psi_{\mathrm{prod}}\rangle$ implies persistent quantum correlations despite environmental interaction.

The mixed states $\rho_{1}$ and $\rho_{2}$ behave similarly to the product states; however, with a stronger synchronicity during the evolution. Their synchronous behavior rests in their higher purity than the product states; however, their complete loss of quantum discord into the classical realm indicates that, however pure, their combination generates classical relations for their substates (Figure 5).

From Figure 5, we see a further confirmation that the period t=[1,2] is the critical transition, which delineates that the system is moving towards quantum-like information exchange by increasing the quantum discord for the product states and inducing a slower rate of negative change in the discord for the Bell state and the mixed state $\rho_{1}$. Indeed, this is equally well confirmed by the rate of change in the entanglement entropy of all the states, as an indicator of whether a system moves towards quantum-like information exchange or classical information exchange.

### 3.5. Entropy and Entanglement Entropy by Parametrized Coefficients of Mixed States

The goal of this final part of the numerical analysis of the GKSL equation is to study the camel-hump-landscape defined by Definition 1 for the entropy and entanglement entropy for mixed quantum states parameterized by ϕ under Lindblad evolution. As shown in the Methods section, mixed states are constructed as convex combinations of pure states, where the coefficients α,β,γ, and δ are defined by trigonometric functions of ϕ (see Method section). The mixed state that is considered for this final calculation is defined as

(32) $$\rho_{0}=0.5|\psi_{1}\rangle \langle \psi_{1}\left|+0.5\right|\psi^{\prime }\rangle \langle \psi^{\prime }|,$$

where the pure states $|\psi_{1}\rangle$ and $|\psi^{\prime }\rangle$ are given by

(33) $$|\psi_{1}\rangle =\alpha |00\rangle +\beta |01\rangle ,\;|\psi^{\prime }\rangle =\gamma |10\rangle +\delta |11\rangle .$$

So the convex combination of these two pure states forms the mixed state as

(34) $$\rho_{0}=0.5(\alpha |00\rangle +\beta |01\rangle )(\alpha^{*}\langle 00|+\beta^{*}\langle 01|)+0.5(\gamma |10\rangle +\delta |11\rangle )(\gamma^{*}\langle 10|+\delta^{*}\langle 11|).$$

These states evolve according to the Lindblad master equation, governed by the camel-like evolution Hamiltonian as given by [2]. The von Neumann entropy, S(ρ)=−Tr(ρlogρ), is calculated at each time step to quantify the uncertainty or mixedness of the state. By varying ϕ∈[−π,π], the dependence of steady-state entropy on the parameterization is analyzed, providing insight into how the structure of the coefficients influences the quantum system’s dynamics.

**Figure 6:**
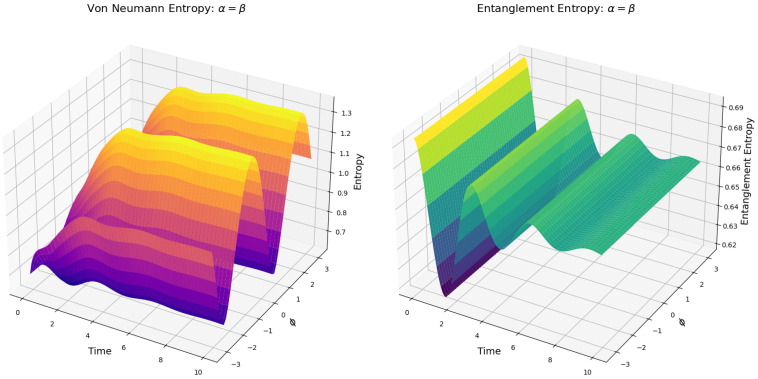
**Left**: Entropy evolution. **Right**: Entanglement entropy evolution. System simulated by the parametrization of the convexity coefficients, for the mixed state $\rho_{0}$, with p=q=0.5. $\gamma =\frac{1}{2}$ under the Khrennikov-picture [2]. Here, we plot the entropy evolution for α=β from equation (34). The case of β is identical to α, so it is omitted for graphical purposes. The simulation was initiated with all pairs of states entangled (not separated) at t=0.

From Figure 6 (left), we see that the entropy dynamics observed in the simulations reveal a periodic behavior with a period of 2π determined by the trigonometric parametrization of the coefficients α,β,γ, and δ using cos(ϕ) and sin(ϕ). This periodicity, inherent to the symmetry of the parameterization, is reflected in the steady-state entropy values, which exhibit maxima at ϕ=−π/4 and ϕ=3π/2, and minima at ϕ=π/4 and ϕ=−3π/2, accordingly with the nature of $\cos^{2}\phi$ and $\sin^{2}\phi$ by the normalization condition $\cos^{2}\phi +\sin^{2}\phi =1$. These points correspond to configurations where the quantum system achieves the highest and lowest levels of mixedness, respectively (Figure 6).

Figure 6 (left) shows that the periodic high-entropy states yield an increased uncertainty in the system, while the low-entropy states imply closer alignment to pure states (compare with Figure 2). However, a key insight is that the camel-like landscape gradually vanishes as the entropy grows by the periodic change in the value of ϕ (Figure 6, left). By considering Definition 1, we see in Figure 6 (left) that as the value of the coefficients evolves towards ϕ=−π/4 and ϕ=3π/2 as the transition state. This implies that the transition state is no longer a transition state followed by a relaxed steady state but a step towards a steady state of higher entropy than the transition state itself. By this, we can conclude that the camel-like behavior of the entropy given by [2] given in Figure 2 is sensitive to the parametrization of the coefficients of the quantum states.

Nevertheless, we find an equally interesting result in the evolution of the entanglement entropy (Figure 6, left). The parametrization of the quantum state coefficients by ϕ has no effect on the entropy of the reduced density matrices. This is expected, as entanglement entropy depends only on the structure of the total state and not on any direct interaction between $\psi_{1}$ and $\psi^{\prime }$. As a result, the entanglement entropy for the mixed states follows the same transition-state-based landscape as when the coefficients are fixed (Figure 3).

We can now construct the following theorem, relating the behavior of the entropy, entanglement entropy, the rate of information exchange, and the quantum discord in Figure 2, Figure 3, Figure 4 and Figure 5.

**Theorem** **1** (Classical versus non-classical evolution). *Let ρ(t) be the density matrix of a quantum system at time t, and let $S_{E}\left(t\right)$ denote the entanglement entropy of a bipartite subsystem A∪B of the system, where $S_{E}\left(t\right)=-Tr\left(\rho_{A}\left(t\right)\log\rho_{A}\left(t\right)\right)$ and $\rho_{A}\left(t\right)={Tr}_{B}\left(\rho \left(t\right)\right)$ is the reduced density matrix of subsystem A. Let furthermore J be the entire period of the evolution of the states under a completely positive trace-preserving (CPTP) map, and let I⊂J.*

***Assume the following**:*

*1.*  *The system evolves under a completely positive trace-preserving (CPTP) map (i.e., the GKSL equation), which ensures the physicality of the evolution.*
*2.*  *The entanglement entropy $S_{E}\left(t\right)$ is differentiable with respect to time t.*

***Then, it follows**:*

*1.*  *If $\frac{dS_{E}\left(t\right)}{dt}<0$ for all t in some interval I, the system’s information exchange evolves towards classical-like behavior in I. This implies a reduction in quantum correlations, such as entanglement, and a shift towards classical correlations, where the system behaves more classically.*
*2.*  *If $\frac{dS_{E}\left(t\right)}{dt}>0$ for all t in some interval I, the system’s information exchange evolves towards quantum-like behavior in I. This implies an increase in quantum correlations, such as entanglement, which enables non-classical phenomena like quantum teleportation.*
*3.*  *The measured substate shifts the quantum discord toward 0 by adding classical contributions, while the unmeasured substate shifts it toward 1 if $\frac{dS_{E}}{dt}>0$, and conversely if $\frac{dS_{E}}{dt}<0$.*

**Proof.**  Since the system evolves under a CPTP map (completely positive trace-preserving map), the density matrix ρ(t) evolves as $\rho \left(t\right)=E_{t}\left(\rho \left(0\right)\right)$, where $E_{t}$ is a CPTP map. This guarantees that $\rho_{A}\left(t\right)$ remains a valid density matrix. Furthermore, we base our proof on the universal relation:

(35) Bell-nonlocality⊃Quantumsteering⊃Quantumentanglement⊃Quantumdiscord

The entanglement entropy $S_{E}\left(t\right)$ is defined as the von Neumann entropy of the reduced density matrix $\rho_{A}\left(t\right)$:

$$S_{E}\left(t\right)=-\mathrm{Tr}\left(\rho_{B}\left(t\right)\log\rho_{B}\left(t\right)\right).$$

By assumption, $S_{E}\left(t\right)$ is differentiable, and we analyze the implications of $\frac{dS_{E}}{dt}$ and prove **points 1 and 2**.

**Case 1:** If $\frac{dS_{E}\left(t\right)}{dt}<0$ for all *t* in some interval *I*, this implies that the entanglement entropy is decreasing over time. Since entanglement entropy measures quantum correlations, its decrease suggests that quantum information is being lost, likely due to decoherence. This results in a reduction in quantum correlations, leaving only classical correlations dominant, signifying classical-like behavior.

**Case 2:** If $\frac{dS_{E}\left(t\right)}{dt}>0$ for all *t* in some interval *I*, then entanglement entropy is increasing. Since increasing entropy indicates an increase in quantum correlations, the system is evolving towards more non-classical correlations, including entanglement and quantum discord (see relevant sections). This suggests that the system’s information exchange is dominated by quantum effects, signifying quantum-like behavior.

**For point 3**, recall that the quantum discord $D_{B}\left(\rho \right)$ quantifies the difference between total and classical correlations after a measurement on subsystem *B*. Measurements on *B* tend to remove quantum correlations associated with *B*, driving discord toward zero by increasing classical contributions. Conversely, the unmeasured subsystem *A* may retain or increase quantum correlations when $\frac{dS_{E}}{dt}>0$, pushing discord toward 1. This behavior follows from the monotonicity of quantum mutual information under the local GKSL equation (see Appendix A), which ensures that local measurements reduce quantum correlations in the measured subsystem. Hence, the sign of $\frac{dS_{E}}{dt}$ governs the direction of discord change depending on which substate is measured. □

**Remark** **1.**

*1.*  *The theorem assumes that entanglement entropy is a valid measure of quantum entanglement, which holds for pure and product states, by considering the results from this study. The theorem also holds for mixed states since the parametrization by ϕ of the coefficients of the subsystems does not affect the entanglement entropy landscape.*
*2.*  *The theorem applies to bipartite systems. For multipartite systems, the behavior of entanglement entropy can be more complex, and additional considerations may be necessary.*
*3.*  *The theorem assumes differentiable entanglement entropy, which may not hold in all cases (e.g., during quantum phase transitions or in open quantum systems with non-Markovian dynamics or under discrete Markovian quantum dynamics).*

### 3.6. Quantum Topological Analysis of the Camel-like Framework Solutions of the Entropy

We consider the evolution of a two-level quantum system (qubit) under the Gorini–Kossakowski–Sudarshan–Lindblad (GKSL) equation:

(36) $$\rho^{\prime }\left(t\right)=-i\left[H,\rho \left(t\right)\right]+\gamma \left(C\rho \left(t\right)C^{†}-\frac{1}{2}\left\{C^{†}C,\rho \left(t\right)\right\}\right),$$

where ρ(t) is the density matrix of the system, *H* is the Hamiltonian governing unitary evolution, *C* is the Lindblad operator representing system-environment interaction, and γ is a coupling constant controlling the strength of dissipation, which we calculated to $\gamma =\frac{1}{2}$ in [24].

Following [24], we apply

(37) $$H=\sigma_{x}=\left(\begin{array}{cc} 0 & 1\\ 1 & 0 \end{array}\right),\;C=\left(\begin{array}{cc} 0 & 1\\ 0 & 0 \end{array}\right),\;\gamma =\frac{1}{2}.$$

**Structure of the Density Matrix**

We assume a general initial density matrix of the form:

(38) $$\rho \left(t\right)=\left(\begin{array}{cc} \rho_{11} & \rho_{12}\\ \rho_{21} & 1-\rho_{11} \end{array}\right),$$

where all diagonal entries are real functions, while off-diagonal entries are complex conjugates of one another.

#### 3.6.1. Derivation of the Evolution Equations

Substituting (37) in (36) we solve for each component:

(39) $$-i\left[H,\rho \right]=\left(\begin{array}{cc} i(\rho_{12}-\rho_{21}) & i(2\rho_{11}-1)\\ i-2i\rho_{11} & -i(\rho_{12}-\rho_{21}), \end{array}\right)$$

(40) $$C\rho C^{†}=\left(\begin{array}{cc} 1-\rho_{11} & 0\\ 0 & 0 \end{array}\right),$$

(41) $$C^{†}C\rho =\left(\begin{array}{cc} 0 & 0\\ \rho_{21} & 1-\rho_{11}, \end{array}\right)$$

(42) $$\rho C^{†}C=\left(\begin{array}{cc} 0 & \rho_{12}\\ 0 & 1-\rho_{11}. \end{array}\right)$$

Then, we get the following anti-commutator term:

(43) $$\frac{1}{2}\left\{C^{†}C,\rho \right\}=\left(\begin{array}{cc} 0 & \frac{\rho_{12}}{2}\\ \frac{\rho_{21}}{2} & 1-\rho_{11} \end{array}\right)$$

combining all terms, and we get the system of equations:

(44) $$\frac{d\rho }{dt}=\left(\begin{array}{cc} \frac{1}{2}\left(-\rho_{11}+2i\rho_{12}-2i\rho_{21}+1\right)\; & 2i\rho_{11}-\frac{1}{4}\rho_{12}-i\\ -2i\rho_{11}-\frac{1}{4}\rho_{21}+i\; & \frac{1}{2}\left(\rho_{11}-2i\rho_{12}+2i\rho_{21}-1\right), \end{array}\right)$$

which we solve numerically in the following sections.

#### 3.6.2. Initial Conditions from Parametrization on the Bloch Sphere

We parametrize the pure states using two parameters, representing a curve on the Bloch sphere. To achieve this, we parametrize into the representation of the pure state superposition on the Bloch sphere.

(45) $$|\psi \left(\theta ,\phi \right)\rangle =\cos\theta |0\rangle +\sin\theta e^{i\phi }|1\rangle$$

which has the corresponding density matrix:

(46) $$\rho_{0}=|\psi \left(\theta ,\phi \right)\rangle \langle \psi \left(\theta ,\phi \right)\rangle$$

explicitly this gives the initial density matrix for the numerical calculation by

(47) $$\rho =\left|\psi \rangle \langle \psi \right|=\left(\begin{array}{cc} \cos^{2}\left(\frac{\theta }{2}\right) & \cos\left(\frac{\theta }{2}\right)\sin\left(\frac{\theta }{2}\right)e^{-i\phi }\\ \cos\left(\frac{\theta }{2}\right)\sin\left(\frac{\theta }{2}\right)e^{i\phi } & \sin^{2}\left(\frac{\theta }{2}\right) \end{array}\right).$$

**Figure 7:**
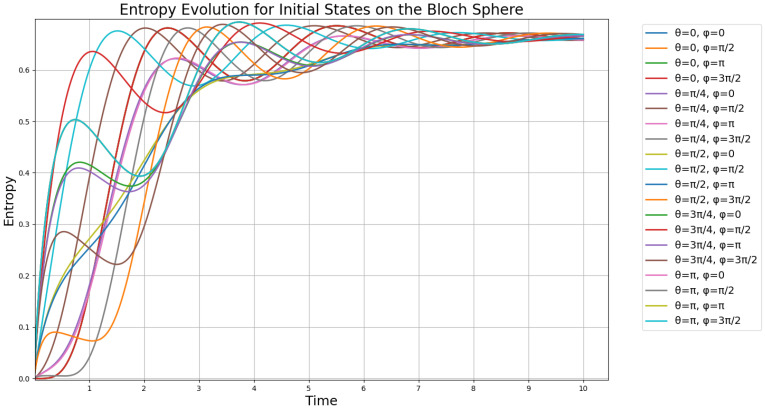
Evolution of the von Neumann entropy of a superposition of pure states with respect to the parameter ϕ on the Bloch sphere in (47), under the GKSL equation in (44) within the camel-like framework.

We proceed to calculate numerical solutions of the GKSL Equation (36) with $\gamma =\frac{1}{2}$ and the operators defined in (16). Using the Bloch sphere density matrix from (47), we vary ϕ and θ and identify the suitable initial conditions by the resulting spectrum of numerical entropy solutions, as shown in Figure 7 as overlaid plots.

**Figure 8:**
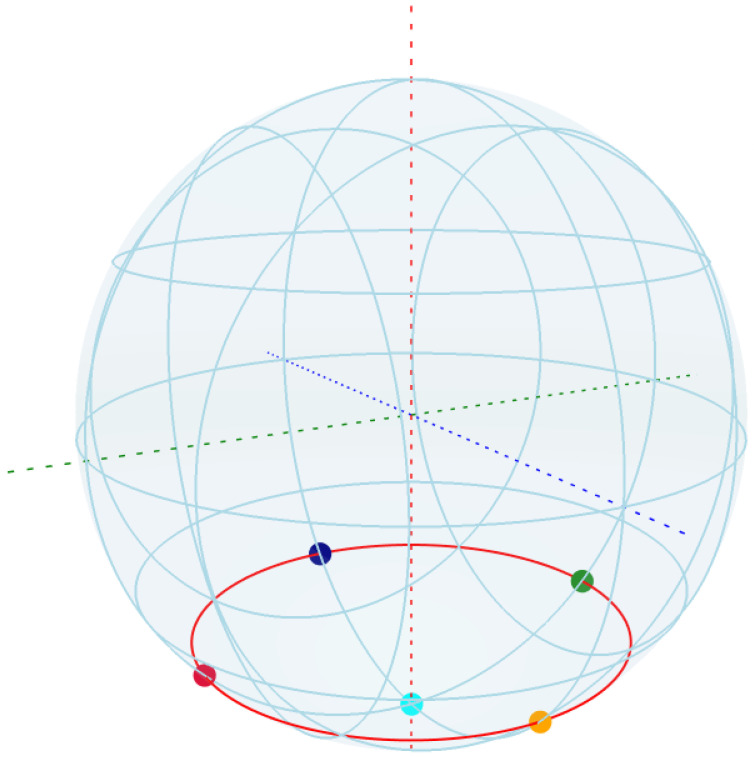
Unstable states by the parameters ϕ and θ from the set S in (48) represented on the Bloch sphere. The four unstable points at each quadrant intersection on the lower quarter circle $\left(\theta =\frac{3\pi }{4}\right)$ are shown across the red circle belt. The South Pole state has multiplicity of 2 and is shown in azure, where both |1〉,−i|1〉) occur, forming six states visible as five decoherence-sensitive states.

From Figure 7, we can see that six unstable states immediately decohere, forming an important class of quantum states with importance for quantum coding purposes. We identify these on the left-most part of the plot and find that these states on the Bloch sphere form the set:

(48) $$S=\left\{\left(\frac{3\pi }{4},0\right),\left(\frac{3\pi }{4},\frac{3\pi }{2}\right),\left(\frac{3\pi }{4},\pi \right),\left(\frac{3\pi }{4},\frac{\pi }{2}\right),\left(\pi ,0\right),\left(\pi ,\frac{3\pi }{2}\right)\right\}$$

which gives the specific states:

(49) $$\begin{array}{cc} |\psi (\frac{3\pi }{4},0)\rangle & =\cos\left(\frac{3\pi }{8}\right)|0\rangle +\sin\left(\frac{3\pi }{8}\right)|1\rangle \\ |\psi (\frac{3\pi }{4},\frac{3\pi }{2})\rangle & =\cos\left(\frac{3\pi }{8}\right)|0\rangle -i\sin\left(\frac{3\pi }{8}\right)|1\rangle \\ |\psi (\frac{3\pi }{4},\pi )\rangle & =\cos\left(\frac{3\pi }{8}\right)|0\rangle -\sin\left(\frac{3\pi }{8}\right)|1\rangle \\ |\psi (\frac{3\pi }{4},\frac{\pi }{2})\rangle & =\cos\left(\frac{3\pi }{8}\right)|0\rangle +i\sin\left(\frac{3\pi }{8}\right)|1\rangle \\ |\psi (\pi ,0)\rangle & =|1\rangle \\ |\psi (\pi ,\frac{3\pi }{2})\rangle & =-i|1\rangle \end{array}$$

which are states that are highly unstable towards decoherence and dephasing. We have a total of six such states, where the last two in (49) overlap on the South Pole. In order to find how these states appear on the Bloch sphere, we plot the Bloch sphere with these six states arranged as spherical coordinates (Figure 8).

From the plot in Figure 8, we find that the unstable states arise around the South Pole of the Bloch sphere, in the well-defined third quarter-circle for θ, at each quadrant intersection for the value of ϕ. Notably, the four states on this quarter circle correspond to equal superpositions with distinct relative phases, aligned with the principal axes of the Bloch sphere. These geometrically significant points delineate a partition of the Bloch sphere where entropic instability becomes prominent, particularly through the emergence of rapid decoherence with no oscillatory components, which then decays oscillatory towards the steady state (see Figure 7).

### 3.7. Topological Analysis on the Bloch Sphere

The set S in (48) can be classified topologically on the Bloch sphere $S^{2}$. We establish coordinates on this manifold using the standard spherical parameterization:

(50) (x,y,z)=(sinθcosϕ,sinθsinϕ,cosθ)

This allows us to combine topologically all stable solutions (which decohere slowly under the camel-like framework) as the complement $S^{2}\setminus X$, forming a non-compact topological space homeomorphic to a 2-sphere with 5 punctures, where each puncture corresponds to one of the highly unstable states in S in (48) (recall two of the six states are the same on the Bloch spheres’ South Pole giving a total of 5 punctures). The set S of unstable states in (48) can be interpreted as the zero-set of a map

(51) $$f:S^{2}\to R^{2}$$

where *f* vanishes precisely at these six specific points. We can express this map as

(52) $$f\left(\theta ,\phi \right)=(g_{1}\left(\theta ,\phi \right),g_{2}\left(\theta ,\phi \right))$$

where $g_{1}$ captures the pole structure (with zeros at θ∈{0,π/4,π}), and $g_{2}$ represents the angular dependence (with zeros at specific ϕ values depending on θ). Here, we can form the function:

(53) $$\begin{array}{cc} g_{1}\left(\theta \right) & =\left(\cos\theta +\frac{\sqrt{2}}{2}\right)\cdot \left(\cos\theta +1\right)\\ g_{2}\left(\theta ,\phi \right) & =\sin^{2}\left(\phi \right)\cdot \sin^{2}\left(\phi -\frac{\pi }{2}\right)\cdot \left(\cos\theta +1\right) \end{array}$$

This function vanishes precisely at the eight points of set S in (48). The component $g_{1}$ captures the latitude dependence, vanishing at $\theta \in \{\frac{3\pi }{4},\pi \}$, while $g_{2}$ encodes the longitude constraints, vanishing at the South Pole and at four specific longitudes $\phi \in \{0,\frac{\pi }{2},\pi ,\frac{3\pi }{2}\}$ when $\theta =\frac{3\pi }{4}$. The factor (cosθ+1) in $g_{2}$ ensures it vanishes at the South Pole regardless of ϕ. The reason we construct this function which vanishes for the unstable points selected from Figure 7 is based on forming a topological analysis of where the stable states arise, which are states that satisfy a gradual decoherence and dephasing under the camel-like framework. These decoherence-instable states in the set (48) are indeed still valid as solutions to the GKSL equation; however, as they decohere immediately, they form a special case of states that pose specific consequences for quantum computation purposes, as we briefly mentioned above. Therefore, isolating these states on the sphere can give us a topologically intuitive view of how states develop stability and instability to decoherence effects by the dissipation operator in the GKSL equation.

Thus, the function (53) characterizes the set S of the unstable states as the zero set, where $f\left(\theta ,\phi \right)=(g_{1}\left(\theta \right),g_{2}\left(\theta ,\phi \right))=\left(0,0\right)$, while (more) stable states correspond to points where f(θ,ϕ)≠(0,0). We can use this framework on the Bloch sphere to start by performing a gradient flow analysis to study the evolution of states as they approach the highly unstable states in S on the Bloch sphere.

#### 3.7.1. Gradient Flow Analysis on the Bloch Sphere

**Figure 9:**
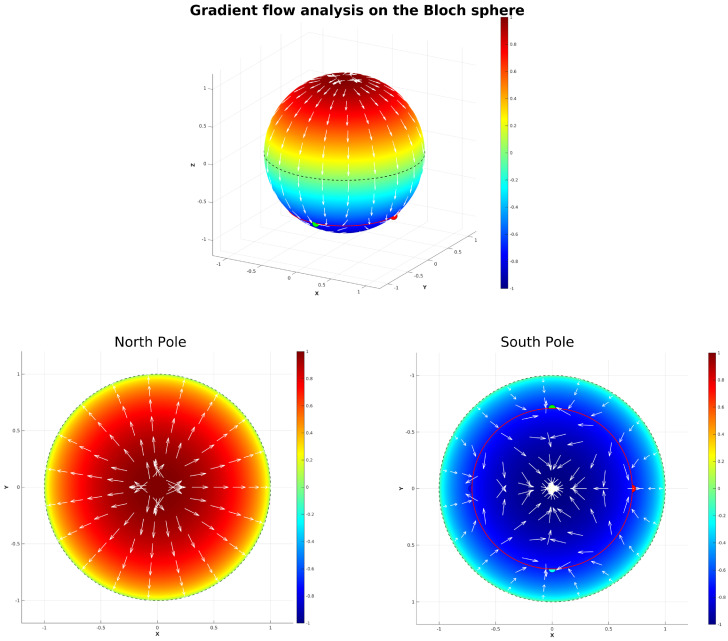
The gradient flow of the Lyapunov function $h\left(\theta ,\phi \right)=g_{1}{\left(\theta \right)}^{2}+g_{2}{\left(\theta ,\phi \right)}^{2},$ where $g_{1}$ and $g_{2}$ are defined in (53). Red points: Highly unstable points from the set S defined in (48). The circle highlights the third quarter circle θ=3π/4, where states are highly unstable. White arrows indicate the gradient flow direction, while cyan lines show specific flow trajectories. Blue regions represent areas where h(θ,ϕ) is close to zero and where the states are unstable and decohere rapidly. Red regions represent areas where *h* has large values and where the inherent states are stable and do not decohere rapidly. All states on the surface have maximal purity, being on r=1 on the Bloch sphere.

The function

$$h\left(\theta ,\phi \right)=g_{1}{\left(\theta \right)}^{2}+g_{2}{\left(\theta ,\phi \right)}^{2}$$

where $g_{1}$, $g_{2}$ are defined in (53) is constructed as a scalar potential function (or a Lyapunov-like function) on the Bloch sphere, $S^{2}$, whose global minima are precisely the five unstable points in the set S in (48). These minima occur at the four points on the three-quarter circle ($\theta =\frac{3\pi }{4}$) with $\phi \in \{0,\frac{\pi }{2},\pi ,\frac{3\pi }{2}\}$, and at the South Pole (θ=π). By computing the negative gradient, −∇h, we define a gradient dynamical system $\frac{d}{dt}\left(\theta ,\phi \right)=-\nabla h\left(\theta ,\phi \right).$ The integral curves (trajectories or flow lines) of this vector field represent paths of steepest descent on the potential surface *h*. Plotting these trajectories visualizes the basins of attraction for each of the five special points under this gradient flow. This analysis provides topological insights into the phase portrait of the original system by partitioning the Bloch sphere according to the influence of these unstable points and their effect on solving the system using the ODE45 method. By visualizing the trajectories that follow $\frac{d}{dt}\left(\theta ,\phi \right)=-\nabla h\left(\theta ,\phi \right)$ on the Bloch sphere, we can identify the resulting gradient field structure, where the set S of unstable points forms a $C_{4}$ symmetry in the ϕ direction that is preserved by the gradient flow due to the periodic structure of $g_{2}$. This partitioning reveals how the Bloch sphere is divided into the two distinct regions, Northern and Southern hemisphere, where quantum states in each region are attracted to their corresponding decoherence hotspot, either the South Pole, or the four concentric points on the Braiding ring ($\theta =\frac{3\pi }{4}$ (see Figure 9)).

The flow lines in Figure 9 indicate that unstable points near $\theta =\frac{3\pi }{4}$ act as attractors for certain states descending from the North Pole. As shown in the figure, the gradient structure around the South Pole reveals a complex flow pattern towards the attractor at the South Pole. The deep red region near the North Pole, corresponding to the highest gradient values, shows that decoherence and dephasing drive states as a source toward the South Pole, while the gradient flow (white arrows) reveals this as the dominant evolution pathway. The gradient lines evolve globally away from the third quarter circle ($\theta =\frac{3\pi }{4}$), exhibiting bidirectional flow, toward the South Pole (dominant pathway), and back toward the North-West and North-East near four specific points on the third quarter circle. This behavior, clearly visible in Figure 9 (South Pole image), reveals that the third quarter circle acts as a topological transition region, by the specific Lyapunov function. The gradients around both the South Pole and this quarter circle show bifurcating flows, creating what may be described as an open barrier that introduces disorder in the evolution of states.

These observations demonstrate a topological tendency of the solutions of the GKSL equation where the third quarter-circle at $\theta =\frac{3\pi }{4}$ acts as a transition region and the states can evolve both slightly Northward and due Southward. The system thus exhibits a topological transition containing unstable states and trajectories, with gradient-driven evolution creating complex flow patterns around these selected unstable points in (48).

It is worth noting that these critical points simply emerge from the global shape of *h* and do not affect the form of the gradient field itself. The “camel-like” geometry of the entropy thus arises solely from the interplay of the Hamiltonian and dissipative terms encoded in *h*, rather than from any special, localized influence of the unstable points. This implies that the gradient flow trajectories around the unstable point are also real in a physical experiment, as long as we consider the points S in (48) as “special” or “different”, and hence, are evaluated in *h* by being outside the desired stability against decoherence of states under the camel-like framework.

Finally, we note that the trajectories on the Bloch sphere represent a multitude of different transitions between pure states under an entirely unitary evolution, as they remain on the surface of the Bloch sphere. In a physical case, this would be represented by a series of diffractometers for beamed photons, which preserves the purity while changing their polarization. Conclusively, in this section, we have thus constructed a Lyapunov function based on five highly unstable quantum states under the camel-like framework by their entropy plot in Figure 7, in order to design an trajectory path for the evolution of states under a completely unitary and real experiment in quantum information, so that we can predict the evolution of quantum states under the camel-like framework. It is also worth noting that the North Pole represents the most stable point in terms of decoherence resistance, and the South Pole represents the most unstable. By the gradient field we have designed here, the instability of states is thus not related to instability/stability towards decoherence but stability and instability based on the gradient flow values that −∇h attains on the Bloch sphere, which are useful for the mapping we have presented in Figure 9.

#### 3.7.2. Topological Constraints on the Bloch Sphere

With the notion that the North Pole acts as a source and the South Pole as an attractor by considering the gradient field dynamics, we can form a basic basin map and analyze the evolution of the stability of the quantum states, subdivided into topological basins. We develop this analysis from the quantum system’s fundamental functions given in (53). The gradient flow dynamics are derived from the potential function $h\left(\theta ,\phi \right)=g_{1}{\left(\theta \right)}^{2}+g_{2}{\left(\theta ,\phi \right)}^{2}$, with the evolution equations

(54) $$\begin{array}{cc} \frac{d\theta }{dt} & =-\frac{\partial h}{\partial \theta }\\ \frac{d\phi }{dt} & =-\frac{1}{\sin\theta }\frac{\partial h}{\partial \phi } \end{array}$$

which we derive from the Riemann flow on the $S^{2}$ sphere [35]. Following these dynamics, we classify the quantum state space into distinct basins:

(55) $$\mathrm{Basin}\left(\theta ,\phi \right)=\left\{\begin{array}{cc} \mathrm{BASIN}1, & \mathrm{if}\;\theta <\frac{3\pi }{4}\\ \mathrm{BASIN}2, & \mathrm{if}\;\frac{3\pi }{4}<\theta \leq \pi \end{array}\right.$$

The boundaries of each basin are defined as a manifold with boundary and can thus be viewed as compact topological sets on the Bloch sphere, which can be assigned well-defined closures, and where any calculation will converge within the established boundaries. We can thus define these basins topologically as

(56) $$\partial B=\{\left(\theta ,\phi \right)\in S^{2}|\forall \epsilon >0,\exists \left(\theta_{1},\phi_{1}\right),\left(\theta_{2},\phi_{2}\right)\in B_{\epsilon }\left(\theta ,\phi \right)$$

where $\left(\theta_{1},\phi_{1}\right)\in B_{1}$ and $\left(\theta_{2},\phi_{2}\right)\in B_{2}$. Here, $B_{\epsilon }\left(\theta ,\phi \right)$ represents the ϵ-neighborhood around the point (θ,ϕ), and $B_{1},B_{2}$ are distinct basins. The unstable points (in terms of decoherence) of the system given in (48) are characterized by:

(57) $$\mathrm{Unstable}\;\mathrm{points}=\{\left(\theta ,\phi \right)\in S^{2}|g_{1}\left(\theta \right)=0\;\mathrm{and}\;g_{2}\left(\theta ,\phi \right)=0\}$$

**Figure 10:**
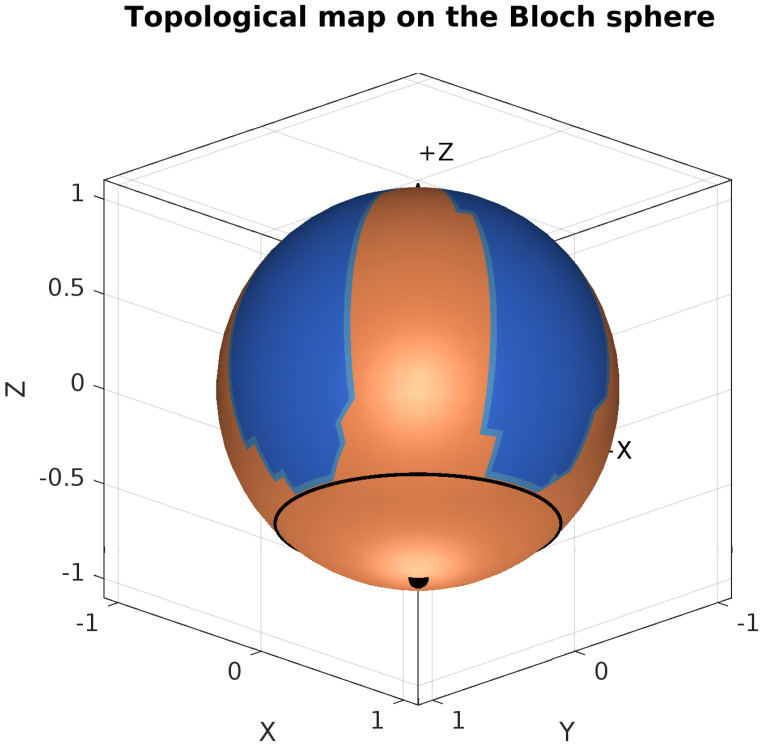
The Bloch sphere colored by basin classification from the gradient flow analysis. The blue regions contain stable states that evolve towards meta-states, in the southward direction. The red region contains stable and metastable states that evolve towards the South Pole, crossing the third quarter circle.

We plot the subdivision of basins on the Bloch sphere in Figure 10 based on this Riemannian flow geometry, where the topological ridge sinks toward the third quarter circle on periodic longitudes, where we have the Braiding ring, composed of unstable states. This gives two basins, displayed in red and blue, where field lines move towards the South Pole from the North Pole, north of $\theta =\frac{3\pi }{4}$.

In Section 3.7.4, we shall discuss the physical implications of this topological manifold. Meanwhile, we form a theorem summarizing the results from topological analysis of the solutions of the GKSL equation under the camel-like framework.

**Theorem** **2** (Global Convergence on the Bloch Sphere)**.** *Consider the gradient flow $\dot{x}=-\nabla h$ of the function $h\left(\theta ,\phi \right)=g_{1}{\left(\theta \right)}^{2}+g_{2}{\left(\theta ,\phi \right)}^{2}$ on $S^{2}$, where*

(58) $$\begin{array}{cc} g_{1}\left(\theta \right) & =\left(\cos\theta +\frac{\sqrt{2}}{2}\right)\left(\cos\theta +1\right), \end{array}$$

(59) $$\begin{array}{cc} g_{2}\left(\theta ,\phi \right) & =\sin^{2}\phi \cdot \sin^{2}\left(\phi -\frac{\pi }{2}\right)\cdot \left(\cos\theta +1\right). \end{array}$$

*Then, the following:*

*1.*  *The unstable points in terms of rapid decoherence consist of the critical points by the South Pole (π,ϕ), and four saddle points at $\left\{\left(\frac{3\pi }{4},k\frac{\pi }{2}\right):k=0,1,2,3\right\}$. Also, the North Pole is a critical point, however, it is stable in terms of decoherence.*
*2.*  *The South Pole is a global attractor: for all initial conditions $\left(\theta_{0},\phi_{0}\right)\in S^{2}\setminus \left\{\left(0,\phi \right)\right\}$, the flow converges to (π,ϕ).*

**Proof.** 

**(1)** Critical points satisfy ∇h=0. Since $h=g_{1}^{2}+g_{2}^{2}$, we have $\nabla h=2g_{1}\nabla g_{1}+2g_{2}\nabla g_{2}$. At θ=0: $g_{1}\left(0\right)\neq 0$ but $\partial g_{1}{/\partial \theta |}_{\theta =0}=0$, and $g_{2}\left(0,\phi \right)=0$, giving isolated critical points. At θ=π: both $g_{1}\left(\pi \right)=g_{2}\left(\pi ,\phi \right)=0$ due to the factor (cosθ+1). At θ=3π/4: $\partial g_{1}/\partial \theta =0$ and $g_{2}=0$ when sinϕ=0 or cosϕ=0, yielding four saddle points.
**(2)** Since h(π,ϕ)=0 and h(θ,ϕ)>0 for all θ≠π, the South Pole is the unique global minimum. Furthermore, along any trajectory of the gradient flow $\dot{x}=-\nabla h$, the function *h* decreases:
  $$\frac{d}{dt}h\left(x\left(t\right)\right)=\nabla h\cdot \dot{x}=-{\left|\nabla h\right|}^{2}\leq 0.$$
  This means that *h* always decreases (or stays constant) along trajectories, which can be seen in the gradient plot. Since the sphere is bounded and h≥0 everywhere, *h* must approach some limiting value. The only points where trajectories can stop are where ∇h=0 (the critical points). At the North Pole and saddle points on the third quarter circle, small perturbations cause trajectories to move away (since *h* decreases from these points). However, at the South Pole, where h=0 (the minimum), trajectories cannot decrease *h* further, so they must stop. Therefore, all trajectories eventually reach the South Pole, making it the global attractor. □

**Remark** **2.** *It is important to note that the notion of instability described by the gradient flow differs from the instability observed in the von Neumann entropy plots in Figure 7. The gradient flow instability arises from the rate at which the Lyapunov-like function changes over the Bloch sphere, whereas the decoherence-related instability of the states in the set S is due to their susceptibility to the dissipation operator in the GKSL equation under the camel-like entropy framework, which we precisely observe in Figure 7. The gradient field of the Lyapunov function thus illustrates a descent from decoherence-stable states (North Pole) toward decoherence-sensitive ones (South Pole).*

#### 3.7.3. Evolution by Purity in the Bloch Ball

In this section, we investigate the evolution of pure states on the Bloch ball (where the entire space inside $S^{2}$ is considered as a vector space of solutions), following the properties of the GKSL equation under the camel-like framework. The evolution starts from pure states (on the Bloch sphere—$S^{2}$) to mixed states in the center of the Bloch ball (a dense globular manifold). The evolution on the Bloch ball is tracked to simulate a pair of entangled particles that are passed through a series of diffractometers and electromagnetic fields, rearranging their polarity and reducing their state of purity into a mixed state. Naturally, this process is entirely irreversible due to the uncertainty principle and evolves from the Bloch surface to the center, following the paths allowed by the GKSL equation under a camel-like framework. These paths should not be confused with the gradient paths in the $S^{2}$ manifold from Figure 9, where the gradient field lines determine the evolution paths from pure metastable states to stable pure states (stability in the sense of being an admissible solution to the GKSL equation).

**Figure 11:**
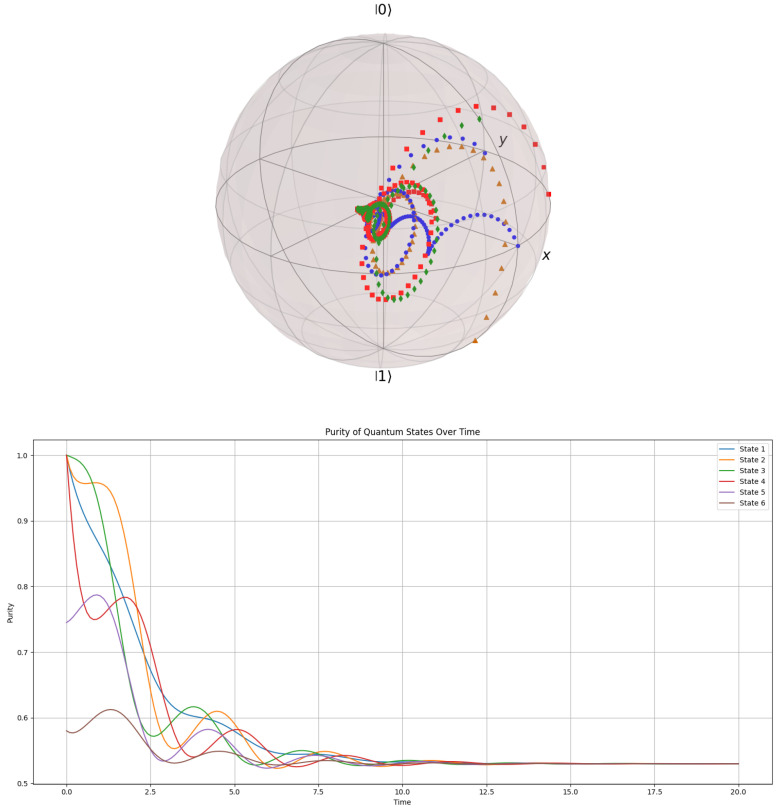
Evolution of quantum states on the Bloch ball by purity. Upper half shows the paths on the Bloch ball from the surface (maximal purity) to the core (mixed states) of six different pure states evolving by the GKSL equation under the camel-like framework into a universal final mixed state. The final mixed state is the convergence steady state for all states on the Bloch sphere under the GKSL equation with the camel-like formalism.

The results are shown in Figure 11 below.

The universal steady state, the great attractor of all states under the GKSL equation in the camel-like framework, is computed in Python and presented as the density matrix shown in Figure 11.

(60) $$\rho_{Universal}=\left(\begin{array}{cc} 0.515041 & 0.00134759+0.121413i\\ 0.00134759-0.121413i & 0.484959. \end{array}\right)$$

#### 3.7.4. Relevance for Physics

The Lyapunov function with four singularities on an unstable equilibrium circle represents a geometric quantum control framework that actively avoids the most decoherence-prone states on the Bloch sphere. These four points at θ=3π/4 are fully admissible pure quantum states, but they exhibit the fastest decoherence rates in the system, making them the most fragile states to maintain coherence by the notion of stability of states in quantum information experiments [36,37]. By strategically placing singularities of $g_{2}\left(\theta ,\phi \right)$ at these four maximally decohering points, the gradient flow creates a control landscape that steers quantum trajectories away from these vulnerable regions, effectively implementing a decoherence-avoiding quantum control protocol [38]. This approach differs from traditional geometric phase schemes by explicitly incorporating decoherence information into the Lyapunov function design, where the unstable manifold at θ=3π/4 represents the locus of states with maximal environmental coupling [39]. The resulting flow pattern, with its characteristic basin structure, ensures that quantum states rapidly escape the high-decoherence region and converge to the more stable states, thereby minimizing exposure to decoherence throughout the evolution [36,38]. This framework can be experimentally realized using polarization qubits passing through engineered diffractometers, where polarization-dependent diffraction losses implement the Lyapunov function h(θ,ϕ). Specifically, using a first diffractometer, we create the $g_{1}\left(\theta \right)$ dependence through selective polarization, while a second holographic diffractometer implements $g_{2}\left(\theta ,\phi \right)$ by creating null diffraction points (the unstable states we call "singularities") at the four decoherence-prone states [40,41]. The measurement backaction from photons lost to higher diffraction orders drives the remaining zero-order photons along the gradient flow trajectories, effectively steering them away from the fragile states at θ=3π/4 toward the decoherence-protected South Pole [42].

## 4. Conclusions

This study provided novel results of simulating the GKSL equation in modeling open quantum systems, emphasizing an analysis of entropy, entanglement entropy, and its rate of change for the Bell, product, and mixed states. The results reveal the evolution under environmental decoherence, with entropy exhibiting a characteristic “camel-like” behavior, a barrier-landscape framework for the GKSL equation developed in [2] and further elaborated and developed in [24]. Most importantly, these results are based on a thorough analysis of the spectrum of the GKSL operator, where the optimal value of the relaxation factor γ was found, and implemented in the calculations for the open quantum system. We have used topology and differential geometry to study the properties of the GKSL equation under the camel-like framework, on the Bloch sphere. Beyond its theoretical significance, the findings of this study may have practical implications in fields such as quantum computing and quantum thermodynamics. The ability to identify transitions towards a classical or a quantum-like information exchange during a system’s evolution could be useful for error correction in quantum computers or understanding dissipation in quantum heat engines. Future work could explore how the camel-like entropy behavior influences quantum circuits’ stability or thermodynamic efficiency in quantum systems.

## Figures and Tables

**Figure A1:**
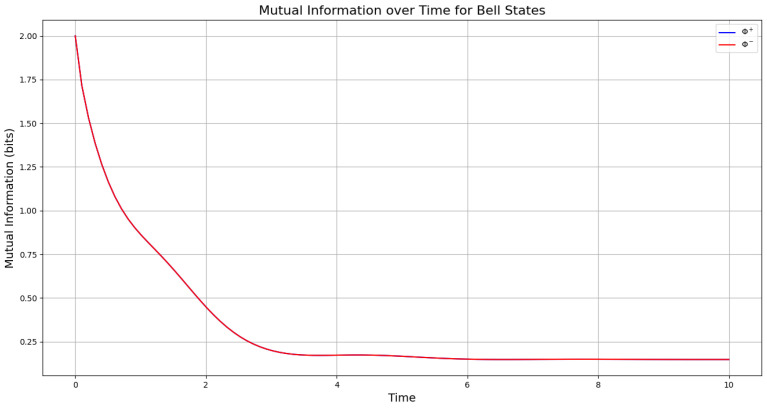

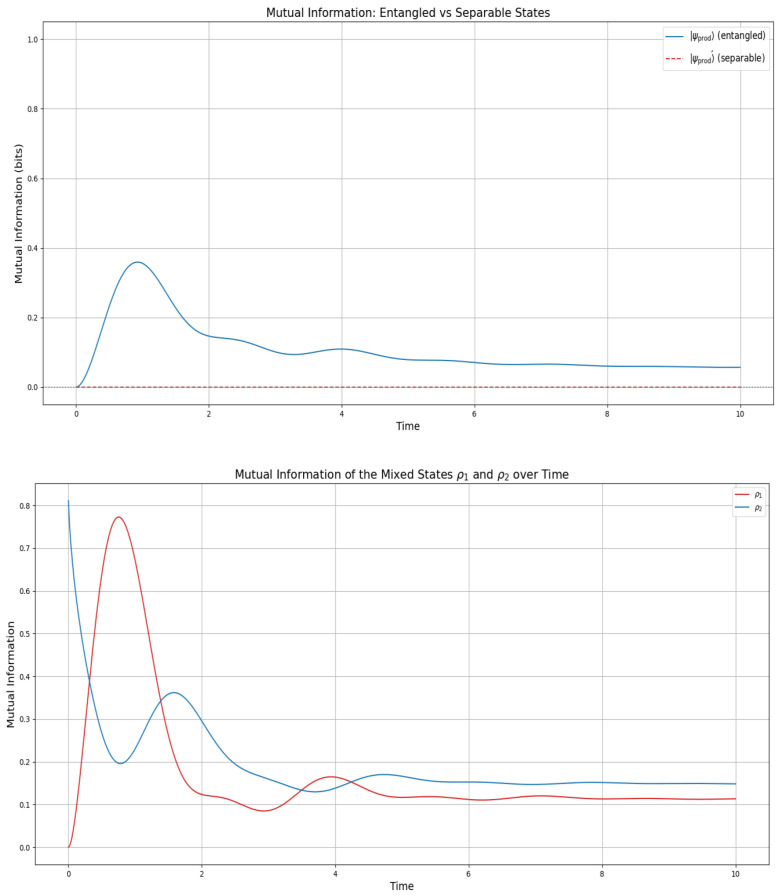
Mutual information of the Bell states $\Phi^{+},\Phi^{-}$ (**top**—overlapping to one plot), the product states in (19) (**middle**), and the mixed states in (23) (with p=0.5 and q=0.5) (**bottom**), calculated by the Lindbladian evolution in the camel-like framework given in [2] with $\gamma =\frac{1}{2}$. The simulation was initiated with all pairs of states entangled (not separated) at t=0. The mutual information is calculated by generating the entropy function using log2, as the mutual information is computed in bits (base-2 units), so it starts at 2 for maximally entangled states, as we see for the Bell states. The mixed state $\rho_{2}$ starts at 0.8, since it is not entirely pure.

## Data Availability

All data can be obtained from the authors via email request.

## Appendix A

### Mutual Information

We study the physical implications that the camel-like framework [2] has for the Bell states, the product states, and the mixed states, with emphasis to Definition 1. For this, we calculate the mutual information, which is a measure of the amount of information exchanged between the states during the Lindbladian evolution of the density matrices. In Figure A1, we see in the top plot the mutual information evolution of the Bell states $|\Phi^{+}\rangle =\frac{1}{\sqrt{2}}(|00\rangle +\left|11\rangle \right)$ (red) and $|\Phi^{-}\rangle =\frac{1}{\sqrt{2}}(|00\rangle -\left|11\rangle \right)$ (blue); initially, both states. Over time, I(A:B) decreases monotonically to 0.15 bits by t=10, driven by decoherence from the Lindblad term with the collapse operator $C=\sigma_{+}⊗I$. Despite this, the entanglement entropy $S(\rho_{A})\approx 1$ bit (see Figure A1) indicates persistent quantum entanglement, maintained by the Hamiltonian $H=\sigma_{x}⊗\sigma_{x}$. The dissipation drives the first qubit toward |0〉, increasing $S(\rho_{AB})$ to 1.75 bits by t≈2, while the second qubit’s entropy decreases to $S(\rho_{B})\approx 0.75$ bits due to asymmetric dissipation. This yields $I\left(A:B\right)=S\left(\rho_{A}\right)+S\left(\rho_{B}\right)-S\left(\rho_{AB}\right)\approx 1+0.75-1.75=0$, though the plot shows a residual 0.1 bits, possibly due to incomplete decoherence. The decline in mutual information suggests that the GKSL framework induces classical-like information exchange, despite persistent quantum entanglement.

The middle plot in Figure A1 compares product states where we have an initially entangled state $|\psi_{prod}\rangle$ (blue) and a separable state $|\psi_{prod}^{\prime }\rangle =|\phi_{A}\rangle ⊗|\phi_{B}\rangle$ (red dashed). For $|\psi_{prod}\rangle$, the mutual information starts at 0 bits, peaks at 0.35 bits around t=1, and stabilizes at 0.08 bits, reflecting a decay of ∼0.3 bits. This behavior arises from the competition between entanglement generation by $H=\sigma_{x}⊗\sigma_{x}$ and correlation loss to the environment at a rate γ=1. In contrast, $|\psi_{prod}^{\prime }\rangle$ remains at 0 bits, confirming the absence of quantum correlations throughout the evolution.

The bottom plot in Figure A1 examines mixed states $\rho_{1}$ (red) and $\rho_{2}$ (blue). For $\rho_{1}$, I(A:B) starts at 0 bits, peaks at 0.75 bits around t=1, and stabilizes at 0.1 bits. For $\rho_{2}$, it begins at 0.8 bits, dips to 0.2 bits, before increasing to 0.35 bits, and also stabilizes at 0.1 bits. Both mixed states exhibit a transition around t∈[1,1.5], with $\rho_{2}$ showing a higher initial mutual information, suggesting stronger initial dependence between subsystems.
